# Selection for Earlier Flowering Crop Associated with Climatic Variations in the Sahel

**DOI:** 10.1371/journal.pone.0019563

**Published:** 2011-05-04

**Authors:** Yves Vigouroux, Cédric Mariac, Stéphane De Mita, Jean-Louis Pham, Bruno Gérard, Issoufou Kapran, Fabrice Sagnard, Monique Deu, Jacques Chantereau, Abdou Ali, Jupiter Ndjeunga, Viviane Luong, Anne-Céline Thuillet, Abdoul-Aziz Saïdou, Gilles Bezançon

**Affiliations:** 1 Institut de Recherche pour le Développement, Montpellier, France; 2 Institut de Recherche pour le Développement, Niamey, Niger; 3 International Center of Research for the Semi-Arid Tropics, Niamey, Niger; 4 Institut National de la Recherche Agronomique du Niger, Niamey, Niger; 5 Centre de Coopération Internationale en Recherche Agronomique pour le Développement, Montpellier, France; 6 Centre Régional AGHRYMET, Niamey, Niger; 7 Université Abdou Moumouni, Niamey, Niger; University of Maribor, Slovenia

## Abstract

Climate changes will have an impact on food production and will require costly adaptive responses. Adapting to a changing environment will be particularly challenging in sub-Saharan Africa where climate change is expected to have a major impact. However, one important phenomenon that is often overlooked and is poorly documented is the ability of agro-systems to rapidly adapt to environmental variations. Such an adaptation could proceed by the adoption of new varieties or by the adaptation of varieties to a changing environment. In this study, we analyzed these two processes in one of the driest agro-ecosystems in Africa, the Sahel. We performed a detailed study in Niger where pearl millet is the main crop and covers 65% of the cultivated area. To assess how the agro-system is responding to recent recurrent drought, we analyzed samples of pearl millet landraces collected in the same villages in 1976 and 2003 throughout the entire cultivated area of Niger. We studied phenological and morphological differences in the 1976 and 2003 collections by comparing them over three cropping seasons in a common garden experiment. We found no major changes in the main cultivated varieties or in their genetic diversity. However, we observed a significant shift in adaptive traits. Compared to the 1976 samples, samples collected in 2003 displayed a shorter lifecycle, and a reduction in plant and spike size. We also found that an early flowering allele at the *PHYC* locus increased in frequency between 1976 and 2003. The increase exceeded the effect of drift and sampling, suggesting a direct effect of selection for earliness on this gene. We conclude that recurrent drought can lead to selection for earlier flowering in a major Sahelian crop. Surprisingly, these results suggest that diffusion of crop varieties is not the main driver of short term adaptation to climatic variation.

## Introduction

Feeding nine billion people in the next few decades is a major challenge [1]. The problem of potential food shortage is particularly acute in developing countries where population growth is the highest. In developing countries, farming communities represent a large proportion of the population who ensure their food security by producing their own food [2]. In developing countries, agriculture depends to a great extent on rainfall conditions, and unfavorable changes in environmental conditions have a direct impact on food security [3]. As a consequence, the impact of climate change on food security is expected to be the highest in developing country [3]–[5]. Faced with this challenge, several studies have highlighted the need for agriculture to be adapted to future conditions [3]. Sub-Saharan Africa is expected to be one of the most susceptible areas [4]. Among Sub-Saharan regional climate forecasts, the Sahel is the region where uncertainty is the highest [6], and models give drastically contrasted outcomes: a major increase or a major decrease in annual rainfall [6]–[7]. However, a more recent study on the Sahel suggests a common phenomenon across climatic models: a delay in the onset of the rainy season and a likely shortening of the rainy season [8]. Thus major uncertainty still characterizes forecasts concerning climate variation in the Sahel. Better forecasts of the impact of climate variation on agriculture will need fine regional climate models, but also a better knowledge of how climate and agriculture interact as well as of how agro-ecosystems respond to climate variation [9]–[10].

**Figure 1:**
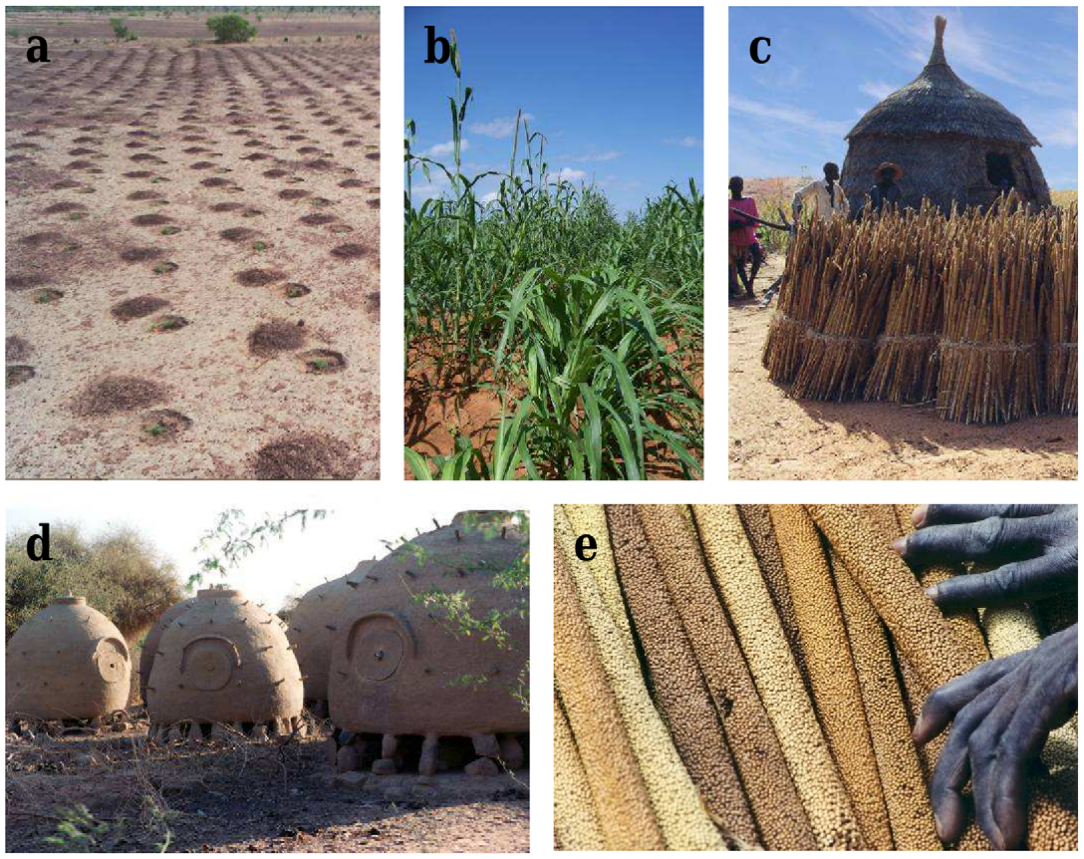
Pearl millet cultivation in Niger. Pearl millet is planted at the beginning of the rainy season after a major rainfall (a). Different traditional varieties exist in Niger and have different flowering phenotypes (b) from early flowering (b, left), to late flowering (b, right). Pearl millet is generally harvested in September or October in Niger (c), bundles of pearl millet spikes are stored in granaries during the dry season (c, d). After harvest, farmers often select the best spike to use as seed in the following planting year (e). Photo by Y. Vigouroux and C. Mariac.

Today, data on the response of agro-ecosystems and of societies to ongoing changes are scarce. For example, could the diffusion or adaptation of varieties already mitigate climatic variations? Understanding this process is of utmost importance as it could provide suitable strategies to reinforce the adaptation of agro-ecosystems. In this study, we addressed this question in the Sahel. The Sahelian agro-ecosystem is dominated by two crops: sorghum and pearl millet. In Niger, pearl millet is the main crop, covering more than 65% of the cultivated area and producing more than 80% of all caloric intake in the country. To understand how traditional cropping systems respond to environmental change, we focused on changes in the evolution and spread of cultivated pearl millet varieties at the national scale. We analyzed the impact of change on the presence of cultivated pearl millet varieties and their adaptation during the drier period that has characterized the Sahel since the early 1970s. Pearl millet is a rainfed crop, and irrigation is rarely used. Farmers sow their fields after the first significant rain which may occur between May and July in Niger (Fig. 1*A*). Several sowing dates are sometimes necessary as high plantlet mortality often occurs because of scarcity of rain at the onset of the rainy season. Different traditional varieties are grown in Niger that vary with respect to spike shape, spike size, grain color and flowering time, among other traits (Fig. 1*B*). Plants are harvested after the end of the rainy season, which is generally in October in Niger (Fig. 1*C*). During the dry season, the bundles of spikes are stored in granaries built in the field or close to the village (Fig. 1*D*). Certain spikes are selected by the farmers and stored to be used as seed in the following growing season (Fig. 1*E*). Sahelian farmers rely mainly on their own seeds, but when there are shortages, they obtain seeds from family members, neighbors or sellers on local markets [11]–[12]. Few modern pearl millet varieties are currently grown in Niger [11].

We used a sample of 192 accessions collected in 1976 throughout Niger [13]. In 2003, a second sampling [14] was performed in the same set of 79 villages covering the entire rainfed cultivated area in Niger (Fig. S1). Phenological and morphological changes in the two samples were compared in a common garden experiment. We also compared changes in the two samples in terms of their genetic diversity at neutral locus and at a quantitative trait locus. We observed a change toward earlier flowering varieties.

## Results

### Characterization of recent climate change in Niger

**Figure 2:**
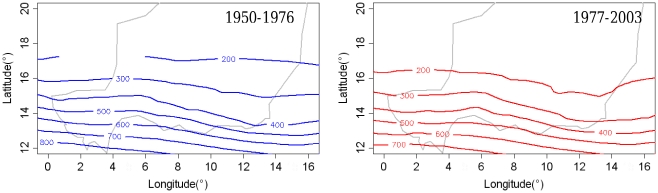
Rainfall isohyets for 1950–1976 and 1977–2003 periods. The rainfall isohyets are plotted for the 1950–1976 periods (blue) and the 1977–2003 periods (red). The shift in isohyets 100 to 150 km further south between the two periods illustrates the average decrease in rainfall.

Sahelian countries have experienced a significant climate shift to drier climate conditions in the last 50 years. At the country scale, we observed a major shift in average isohyetes for the 1977–2003 period compared to 1950–1976 (Fig. 2). The 1977–2003 period was drier than the previous period.

### Changes between 1976 and 2003

The two collections displayed no significant changes in terms of the varieties cultivated [14]. The 1976 sample presented a very good average germination rate (87.2%). The 2003 sample had a slightly higher germination rate (90.0%, t = 1.62, p≤0.044). This minor difference in the germination rate (2.8%) of seeds stored for 27 years at 4°C was evidence that the conservation conditions were very good. We first performed field trials over three years using these seeds.

We first compared the two collections globally. Plants collected in 2003 had a significantly earlier flowering date (F_1,1500_ = 11.4, P≤0.001), were shorter (F_1,1500_ = 40.8, P<0.001), and had shorter spikes (F_1,1500_ = 10.5, P≤0.001) than plants collected in 1976. No significant interactions between sample and field effects were detected but there was a strong significant field effect for each morphological trait: flowering time (F_2,1500_ = 131.9, P<0.001), plant height (F_2,1500_ = 174.3, P<0.001) and spike length (F_2,1500_ = 5.2, P≤0.006).

**Figure 3:**
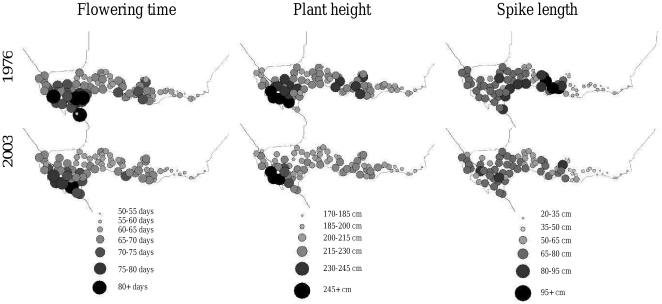
Morphological changes in pearl millet varieties between 1976 and 2003. The average values of morphological and phenological traits are plotted for each of the 79 villages sampled throughout Niger. Significant variations were observed between villages, i.e. varieties collected in 2003 flowered earlier, were shorter and had shorter spikes.

We also performed a statistical analysis of the geographical origin of the varieties. To do so, we first calculated an average flowering time, spike length and plant height for each sampling point based on all the varieties collected in a particular village (Fig. 3). Then we compared the 1976 and 2003 samples by comparing the average values using a paired Wilcoxon test based on their geographical origin. To combine the three field trials into a single test, we used a Fischer combining probability test. Taking geographical origin into account, there was a significant shift in flowering time (Fisher's combined probability, χ^2^ = 42.0, n = 6, P<0.001), plant height (Fisher's combined probability, χ^2^ = 58.9, n = 6, P<0.001), and spike length (Fisher's combined probability, χ^2^ = 47.4, n = 6, P<0.001). The decrease in flowering time and plant height was greatest in the southeastern and central parts of Niger (Fig. 3).

These initial analyses of phenological and morphological changes over time did not distinguish between two phenomena: expansion or contraction of the distribution area of some varieties and possible phenotypic changes in the varieties *per se*. The main reason is that a different number of varieties were collected in 1976 and 2003 in each village. As a consequence, changes in the average value of some traits (e.g. flowering date) could be due to a change in the frequency of certain types of varieties (e.g. early varieties), instead of reflecting a phenotypic change in the variety *per se*. To assess whether variety evolution did play a role in the observed changes, a further analysis was conducted considering only varieties that were sampled in the same village in 2003 and in 1976. Each variety sampled in 2003 was compared with the “same” variety sampled in 1976. Again there was a significant shift for all traits: the 2003 samples displayed a shorter flowering time: 1.2 days shorter (Fisher's combined probability, χ^2^ = 53.4, n = 6, P<0.001), a shorter plant: 6.6 cm shorter (Fisher's combined probability, χ^2^ = 61.8, n = 6, P<0.001) and a shorter spike: 4.5 cm shorter (Fisher's combined probability, χ^2^ = 48.7, n = 6, P<0.001).

All these analyses were based on seed accessions sampled in 1976 and 2003. To assess if our results are robust to this potential protocol bias, we performed several complementary analyses. In the following paragraphs, we assess if a possible seed conservation effect or an increase in earlier flowering weedy plants in the 2003 sample could partially explain the morphological difference observed between samples.

### Analysis of maternal, sampling year and conservation effects

A new field trial analysis was performed using seeds produced in the same field in 2004 from the 2003 and 1976 samples. These samples are hereafter denoted 2003_P2004_ and 1976_P2004_ to differentiate them from the original samples. The germination rate of the seeds produced in 2004 did not present any significant difference between the 1976_P2004_ and 2003_P2004_ samples (t = 1.16, n = 100, p = 0.25). The average germination rate of the 2003_P2004_ and 1976_P2004_ samples was 87.6% (SE ±0.021) and 84.4% (SE ±0.024), respectively. The average seed weight of the 2003_P2004_ sample was 0.978 (SE ±0.006) and of the 1976_P2004_ sample, 0.971 (SE ±0.010). There was no difference in weight between the 1976 and 2003_P2004_ samples (t = −0.60, n = 607, p = 0.55).

A new field experiment was performed in 2007 using these seeds to compare the 1976_P2004_ and 2003_P2004_ samples. The comparison of the 1976_P2004_ and 2003_P2004_ samples revealed highly significant differences in flowering time (F_1,1806_ = 19.6, P<0.001), plant height (F_1,1806_ = 23.3, P<0.001) and spike length (F_1,1806_ = 9.4, P≤0.002) between samples. A significant block effect (F_2,1806_ = 5.3, p≤0.005) was detected for flowering time only, but there was no interaction between blocks and samples (F_2,1806_ = 0.015, P = 0.985). No other block effect or block/sample interactions were detected for the other traits, i.e. plant height and spike length. The average difference between the two samples was a shorter flowering time (2.1 days shorter), shorter plant size (6.5 cm shorter) and shorter spike (2.4 cm shorter) for plants grown from seeds in the 2003_P2004_ sample versus those grown from seeds in the 1976_P2004_ sample. Similar results were obtained in the comparison of sampling sites, i.e. there was a significant decrease in average flowering time, (Fisher's combined probability, χ^2^ = 37.0, n = 6, P<0.001), plant height (Fisher's combined probability, χ^2^ = 33.1, n = 6, P<0.001), and spike length (Fisher's combined probability, χ^2^ = 44.1, n = 6, P<0.001). Finally, when we compared the same varieties sampled in 2003_P2004_ and 1976_P2004_ in the same village, there was also a significant difference in flowering time (Fisher's combined probability, χ^2^ = 35.4, n = 6, P<0.001), plant height (Fisher's combined probability, χ^2^ = 14.3, n = 6, P<0.05) and spike length (Fisher's combined probability, χ^2^ = 34.1, n = 6, P<0.001). Using paired varieties, the estimated average change in flowering time was 1.4 days, in plant height, 4.7 cm and in spike length, 3.34 cm. All these results suggest that storage had no major impact on our initial results using seeds collected in the fields.

### Analysis of the number of intra-varietal weedy plants

A second confounding factor was changes in the frequency of weedy pearl millet plants. Weedy plants are commonly found in pearl millet seedlots in Niger [15]. Weedy plants are characterized by shorter ears, thinner stems, branched stems, partial or total shattering phenotype and long bristles (Fig. S2), and earlier flowering. We wanted to assess whether or not there was an increase in the frequency of weedy plants between the 1976 and 2003 samplings. The number of weedy plants (Fig. S3) was higher in the 1976 sample than in the 2003 sample (Field trial 2004, G = 17.3, p<0.009; Field trial 2005, G = 19.7, p<0.004). This result shows there were significantly fewer weedy plants in 2003 compared to 1976.

### Analysis of correlations between morphological traits and environmental data

**Figure 4:**
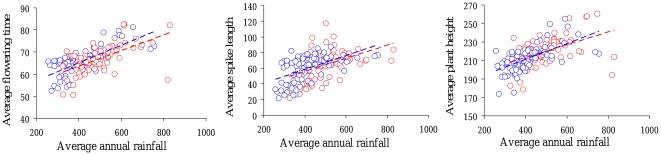
Correlation between average phenological and morphological traits and average annual rainfall. The average flowering time (in days), average spike length (in cm) and average plant height (in cm) for each sampling plot and each year 1976 (red) and 2003 (blue) are plotted against the annual rainfall (in mm). Annual rainfall for 1976 was calculated based on the 1950–1976 period, and for 2003 based on the 1977–2003 period. A significant correlation was observed for flowering time, spike length and plant height for the 1976 samples (r = 0.60, p<0.001; r = 0.46, p<0.001; r = 0.49, p<0.001 respectively) and the 2003 samples (r = 0.72, p<0.001; r = 0.54, p<0.001; r = 0.62, p<0.001 respectively).

For each sampling plot, we also checked if there was a correlation between morphological traits and average rainfall (Fig. 4). We first observed that there was a strong correlation between average rainfall values in the 1950–1976 and 1977–2003 periods (r = 0.99, t = 67.3, n = 79, p<<0.001). There was a significant correlation between average rainfall and flowering time in the 2003 sample (r = 0.72, t = 9.1, n = 79, P<0.001) and the 1976 sample (r = 0.60, t = 6.5, n = 78, P<<0.001). The correlation between average rainfall and spike length was r = 0.54 in the 2003 sample (t = 5.6, n = 79, p<0.001) and r = 0.46 in the 1976 sample (t = 4.6, n = 78, p<0.001). Finally, plant height was also significantly correlated with average rainfall in both the 2003 (r = 0.62, t = 7.0, n = 79, P<0.001) and 1976 samples (r = 0.49, t = 4.6, n = 78, P<0.001). There were no significant correlation differences between 1976 and 2003 in flowering time (t = 1.36, p = 0.17), spike length (t = 0.60, p = 0.54) or plant height (t = 1.17, p = 0.24).

### Genetic comparison of 1976 and 2003 samples

The genetic differentiation (F_ST_) between the two samples (1976 vs. 2003), measured as the difference in allele frequencies at microsatellite loci, was very low (F_ST_ = 0.0015) but statistically significant (P<0.001). Principal component analysis (PCA) revealed slight genetic differences between genotypes in the 1976 and 2003 samples (Fig. S4). The difference between the two samples was not significant on the first PCA axis (Mann-Whitney test, χ^2^ = 3.62, p = 0.057) but was significant on the second axis (Mann-Whitney test, χ^2^ = 5.26, p = 0.022). The average allelic richness was 8.29 (SE 1.32) and 8.39 (SE 1.20) alleles per locus in the 1976 and 2003 samples, respectively. The average genetic diversity was 0.487 (SE 0.052) and 0.488 (SE 0.051) in 1976 and 2003, respectively. The number of alleles (Wilcoxon paired test, Z = −0.37, n = 25, p = 0.71) and genetic diversity (Wilcoxon paired test, Z = −0.21, n = 25, p = 0.83) did not differ between the two samples.

**Figure 5:**
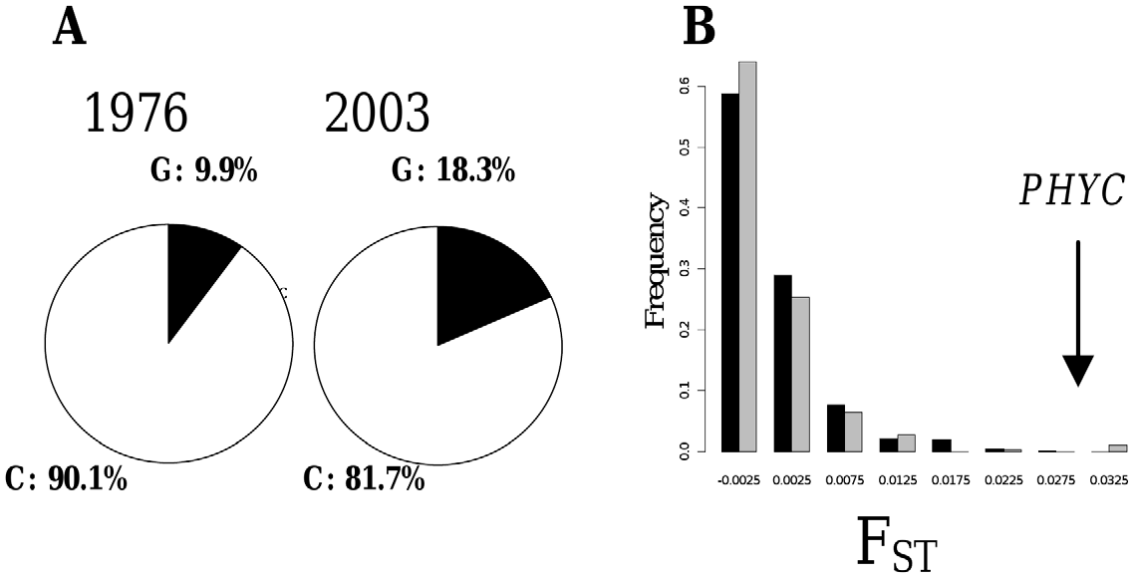
Selection for earlier flowering PHYC alleles. A significant increase (A) in early flowering alleles at the *PHYC* gene (SNP G) was observed between 1976 and 2003. The difference (F_ST_) between the two samples in *PHYC* alleles exceeded the expected effect of drift or sampling (B) based on empirical (dark) or model-based (gray) F_ST_ distributions. These results suggest that selection led to an increase in early flowering alleles at the *PHYC* locus.

Finally, if selection is playing a role in ongoing changes, the frequency of alleles associated with quantitative variation in pearl millet would be expected to change. We tested the change in allele frequency at the flowering locus *PHYC* [16] between the two samples. The earliness allele at the *PHYC* locus increased (G test, p<0.001) from 9.9% to 18.3% between 1976 and 2003 (Fig. 5A).

To assess if the differentiation observed at the *PHYC* locus exceeded the effect of sampling and drift between the two samples, we first constructed an empirical F_ST_ distribution. F_ST_ is generally influenced by a high mutation rate and it might be difficult to compare F_ST_ in markers with different mutation rates: the F_ST_ of microsatellite loci and the F_ST_ of SNPs. Our simulation showed that for the 27 year time frame we considered, the microsatellite F_ST_ was not influenced by the mutation rate, if this mutation rate was lower than 10^−3^ (Fig. S5). The average number of alleles per locus observed in the 1976 and 2003 sample taken together was 10.4. This number of alleles based on our simulations suggests a mutation rate of around 10^−4^ for our set of microsatellite loci (Fig. S5). So the effect of microsatellite mutation rate on F_ST_ is certainly insignificant in the present case.

Finally, to build a model based on F_ST_ distribution, we analyzed population structure. Using STRUCTURE [17]–[18], the likelihood was higher for K = 1 in the 1976 sample and ancestry at K = 2 did not display a clear population structure. The likelihood for K = 2 in the 2003 sample was higher, but no clear genetic group was observed in terms of ancestry (Fig. S6). Using STRUCTURAMA [19] with the 1976 sample, the probability of finding one population in the dataset (X) was Pr[K = 1|X] = 1. With the 2003 sample, Pr[K = 1|X] was 0.9998. So for the two samples, K = 1 was the most likely result. Consequently in subsequent analyses, we considered the two samples to be unstructured. Considering the two populations as unstructured, we estimated the population effective size at 12813 with a 95% lower bound confidence interval of 7248 and a 95% upper bound confidence interval of 34280 (Fig. S7). We used this effective size to build a model based on F_ST_ distribution.

We found that the difference in allele frequency at the *PHYC* locus (Fig. 5B) exceeded the simple effect of drift and sampling based on empirical (P<0.02) or model-based distribution of differentiation (p<0.001). These results suggest a positive selection for the earliness allele.

## Discussion

### Climatic variation and sampling

Western and eastern Africa have experienced a significant decrease in rainfall in the last four decades. The origin of this drier period is assumed to be partially driven by abnormally warmer temperatures in tropical oceans [20]–[21], but also by vegetation dynamics and anthropogenic changes in land use [22]–[23]. Sahel droughts began rather abruptly in the early 1970s [22]–[23]. The initial sample used in this study was collected in 1976, a few years after the serious Sahelian droughts in the early 1970s. This initial sampling was intended to minimize the impact of the droughts [13]. The distribution of varieties in 1976 was very similar to that observed in 2003 [14], and was in agreement with the findings of the distribution study carried out in 1950 [24]. Moreover, only slight overall genetic differences were observed between the 1976 and the 2003 samplings. We stress that sampling is a key component to allow a meaningful comparison of varieties over time. In 2003, we were careful to copy the initial sampling strategy in order to avoid -or at least minimize- the impact of differences in sampling procedures on our results.

### Crop adaptation to climatic variation

Globally there was no statistical difference in the frequencies of the most important groups of varieties in 1976 and 2003 [14]. However, there was a decreasing trend in the relative occurrence of the later flowering variety group from 18.2% in 1976 to 10.2% in 2003 [14]. Some formerly relatively minor varieties were also shown to have a wider geographical distribution in 2003 [14]. The disappearance or spread of varieties certainly played a role in the changes that took place between 1976 and 2003, and was directly shaped by the people's choice of the varieties they cultivated. However, when we compared samples of the same varieties in the same village in 1976 and 2003, we found a significant change, which suggests that some changes in the phenotype of the varieties had actually occurred. We showed that this effect could not be explained by seed conservation effects, an increase in the occurrence of weedy pearl millet plants or sampling effects alone. We also observed a correlation between the phenology of the variety of pearl millet and annual rainfall. Finally, we demonstrated selection for earlier flowering allele at the *PHYC* locus. We suggest that the observed change in annual rainfall is associated with the selection for earlier flowering varieties. Human and environmental constraints certainly paved the way for this adaptation. The variation in flowering time over a period of 27 years was an overall decrease of 1.44 days. Recent climatic ‘middle of the road’ scenarios suggest a shortening of the rainy season by seven days at the end of the 21^st^ century [8]. Thus, by simple extrapolation, a change of 1.9 days in the growing season over 27 years. The adaptation of local varieties could be a significant contribution to the adaptation of varieties to forecast climate changes.

However, adaptation to future climate will depend on the type of selection imposed by the environment [25]. A decrease in rainfall might lead to selection favoring an escape strategy (i.e. rapid flowering) or improved water use efficiency [25]. Recent models suggest that climatic change in the Sahel leads to shortening of the rainy season [8] while total annual rainfall predictions are not very reliable [6]. Rapid flowering or changes in photosensitivity might be an evolutionary road to adaptation to a shorter rainy season. Finally, selection on a particular gene might also be hampered by pleiotropic effects [26]. We actually observed that the earlier flowering *PHYC* allele was also associated with shorter spike length [16]. This earlier flowering allele therefore certainly had an adverse effect on fitness (and consequently an effect on yield). However, this pleiotropic effect did not impair adaptation from 1976 to 2003.

Some recent studies investigated the role that substitution of varieties could play in response to future climate change. Surprisingly, adaptation without substitution in the present case seems to be a significant strategy. One possible reason might be the out breeding reproduction system of pearl millet, which allows strong intra-varietal variability to exist [12]. The strong diversity within a given variety allows adaptation on standing variation. It remains to be determined if the adaptation we observed in pearl millet also applies to selfing Sahelian crops like sorghum. Whatever the case may be, this study illustrated how the diversity in local landraces could be an asset in the response to a variable environment.

## Materials and Methods

### Rainfall data

Isohyets were estimated as previously described [27] for two periods: 1950–1976 and 1977–2003. Isohyets across Niger were built using datasets from the *Centre Regional de Formation et d'Application en Agrométéorologie et Hydrologie Opérationnelle* (AGRHYMET) international research center in Niamey, Niger. The study area was limited to Niger: longitudes 0°–16°E and latitudes 12°N–18°N and concerned 65 rainfall stations. Rainfall estimations for each sampling site were inferred according to their latitude and longitude coordinates, and the isohyets estimated for the two periods. We used the data obtained for the period 1950–1976 and 1977–2003, to estimate the average rainfall for each sampling site.

### Seed collection

Millet landrace accessions were sampled from October 27 to December 26, 1976, in Niger [13]. These samples were stored at 4°C at IRD, Bondy and subsequently at IRD Montpellier, France, without multiplication. Among the 184 villages investigated in 1976, we selected 79 villages. These villages were distributed evenly throughout the country (Fig. S1). A set of 192 different samples from 79 different villages was available from the 1976 initial sampling trip (Fig. S1).

In 2003, a new sampling survey [14] was carried out in the same 79 villages and 420 different accessions were collected (Fig. S1, Table S1). The 2003 sampling scheme was modeled on the 1976 sampling scheme. Dr Borgel, who performed the 1976 sampling, helped design the 2003 sampling scheme. In 2003 and 1976, sampling was carried out in fields or village granaries after harvest. When possible, 30 different spikes were collected from a single farmer population for each variety grown in the village. In 1976, villages with seeds of unknown origin due to complete crop failure in previous years were not sampled [13]. The 2003 samples were stored at 4°C before the July 2004, 2005 and 2007 field trials.

Out of the 192 varieties sampled in 1976, some varieties with the same name and thus sharing common characteristics (spike length, seed color, flowering behavior) were also found in the same villages in 2003. We built a file associating a variety sampled in the same village in 1976 and 2003 based on shared names, and, for some troublesome accessions, on morphology observed in the field. A total of 136 paired varieties were kept (Table S2, Table S3).

We estimated the germination rate of a random sample of 50 accessions from the 2003 collections and 48 accessions from the 1976 collection. A hundred seeds per accession were placed in a petri dish and the number of seeds that had germinated after three days was counted. The germination rate ranged from 0 to 1. To compare the germination rate between samples, we used an arcsin square root transformation of this variable. Differences in the transformed variable were then assessed using a t-test. The average germination rate was 87.2% for the 1976 sample and 90.0% for the 2003 sample. The difference in the germination rate was significant (t = 1.62, p≤0.044), but low (2.8%). This minor difference in the germination rate of seeds stored at 4°C for 27 years shows that the conservation conditions were very good. We also estimated the 100-seed weight of a set of 129 of the 136 paired varieties (Table S2). The two samples were compared (1976 vs. 2003) using a paired Wilcoxon nonparametric test. Seeds from the 1976 sample were significantly heavier than seeds from the 2003 sample (Wilcoxon paired test, n = 129, Z = 3.51, P<0.001). The average 100-seed weights were 1.037 g (SE ±0.015) for the 1976 sample and 0.980 g (SE ±0.013) for the 2003 sample. We next performed a field experiment using these initial seeds.

### Field experiments

Seeds were planted in the field station of the International Center of Research for the Semi-Arid Tropics (ICRISAT), Sadore, Niger. The planting dates were July 7, 2004, July 7, 2005 and June 23, 2007. Plants were sustained by natural rainfall and irrigated if necessary at the beginning or end of the rainy season. For each seedlot accession, 25 plots were sown in 2004 and 2005, and 15 plots in 2007. Around 30 seeds per plot were sown and hand-thinned to three plants two weeks after emergence. Four weeks after emergence, plants were hand-thinned to only one plant. Plants were spaced 0.7 m apart within rows and 0.7 m apart between rows. Field borders were planted with three to six rows of sorghum or pearl millet varieties to avoid side effects. Plants were hand-weeded twice at the beginning of the experiment. Treatments against mildew and insects were performed when needed. In 2004, the experiment was a pseudo-random experiment where villages were randomly selected and then samples collected in 1976 and 2003 for a given village were sown separately. The 2005 and 2007 experiments were completely random experiments where each sampling site and sample was randomly selected. Morphological data of five randomly chosen plants per accession were recorded individually in 2004 and 2005, and of 15 plants in 2007. We recorded the flowering time from planting date to the female flowering stage, plant height and spike length for the three field experiments. In 2004 and 2005, we also recorded the 100-seed weight. In 2004 and 2005, the number of weedy plants out of 25 plants per accession was assessed by two independent observers. The number of accessions with 0, 1, 2, 3, 4, 5 and 6 or more weedy plants was calculated for the 2003 and 1976 samples and for the 2004 and 2005 field trials. The distributions were then compared for significance using a G test [28].

In 2004, five plants of each accession were selfed and individually harvested. This new sample was named 1976_P2004_ and 2003_P2004_. In 2007, these plants were used to perform a new field experiment with three repetitions. The germination rate of these selfed seeds was assessed in 50 random seedlots derived from the 2003 sample and 50 random seedlots derived from the 1976 sample. Each individual's selfed progeny (five per accession) was planted at Sadore ICRISAT field station in June 2007. Three repetitions of the experiment were performed planted on June 15, 2007, June 16, 2007 and June 19, 2007. Days from planting to female flowering, plant height and spike length at maturity were recorded for each individual plant.

### Statistical analysis

To compare morphological differences between the 1976 and 2003 samples, first we calculated an average value for each accession for each field trial (2004, 2005, 2007). We then compared the two samples (1976/2003) using analysis of variance: y_ijk_ = μ+s_i_+b_j_+s_i_*b_j_+ε_ijk_ where y_ijk_ is the phenotypic value, μ is the overall mean, s_i_ is the sample effect (1976/2003), b_j_ is the repetition effect (year or block), s_i_*b_j_ is the sample and repetition interaction effect and ε_ijk_ is the residual error.

For a given sampling site (village), we also compared the differences in phenological and morphological traits. For this purpose, for each sampling site, we calculated an average value for the different morphological traits, i.e. the mean of all varieties sampled in each village. Comparisons between the two sets of sampling sites (1976 vs. 2003) were performed using a paired Wilcoxon nonparametric test for each field trial. Overall significance was obtained using Fisher's combined probability test [28].

A total of 136 varieties sampled in 1976 were found in the same village in 2003. We performed an analysis based on these paired varieties. Average values for each morphological trait (flowering time, plant height and spike length) were compared by a Wilcoxon paired test for each field trial. To obtain an overall test effect across repetitions (field trial for the original seeds or block for selfed seeds), a Fisher's combined probability test was performed [28].

Finally, we calculated a correlation between the previously calculated annual rainfall and the average morphological and phenological traits at each sampling site for the 1976 and 2003 samples, respectively. The correlation was tested using a t-test [28].

### DNA extraction and genetic analysis

For each accession, DNA was extracted from one seed [29]. DNA was distributed in 96-well plates and two negative controls were included (no DNA) on each plate. A set of 25 microsatellite loci were used to genotype the different accessions as previously described [29]–[30]. Ten out of the 25 microsatellite loci we used are already mapped: PSMP2237, PSMP2201, PSMP2206 (linkage group 2), PSMP2216, PSMP2214 (linkage group 3), PSMP2005 (linkage group 4), PSMP2208, PSMP2202, PSMP2202 (linkage group 5) and PSMP2266 (linkage group 7). These microsatellite loci are spread throughout the pearl millet genome. We also genotyped a SNP at the *PHYC* gene [16]. A fragment at the 5′ of the gene was amplified and a restriction assay using PvuII was performed to recognize a C/G SNP (see [16] for primer sequences, PCR and digestion conditions). Genotypes were scored as C/C, G/G and C/G according to their digestion pattern. For microsatellite loci, each PCR product was migrated on an ABIPrism 3100 sequencer and the scoring was manually checked by two different persons. *PHYC* assay was scored on agarose gel [16]. We used allelic richness to compare the number of alleles between the two samples with a different number of individuals. Allelic richness (R) was calculated using the formula:
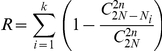
where N_i_ is the number of the i alleles in the population of the largest size N (2N chromosomes), and n is the number of individuals analyzed for the smallest population (2N chromosomes), and k is the total number of alleles at the locus studied [31]. Allelic richness and gene diversity were calculated for the 1976 and the 2003 samples using FSTAT [32] and compared using a non-parametric Wilcoxon paired test. A principal component analysis was also performed on the allele frequencies of each individual. Differentiation (F_ST_) between the two samples was calculated using Power marker [33]. Population structure for the two samples was assessed using STRUCTURE V2.3.1 [17]–[18], based on the 25 microsatellite markers. The burn in period was set at 50000 and the run length at 100000. The number of assumed populations (K) varied from one to five. Five different runs were performed for each K value. The highest likelihood was used for further analyses and ancestry plotting. To assess the number K of populations, we also used STRUCTURAMA [19]. The analysis is based on STRUCTURE [17] but is also possible to sample the number K of populations in a Dirichlet distribution. We used the software with the number K of populations sampled in a Dirichlet distribution, with a mean number of expected populations of five. The output of the analysis is the probability to identify i populations in the dataset Pr[K = i|X]. The analysis was run with 100000 MCMC steps and five chains.

### Analysis of differentiation at the *PHYC* locus

We first calculated the differentiation for each microsatellite allele between the two samples 1976 and 2003 using Powermarker [33]. The distribution obtained is referred to as the empirical distribution. The differentiation obtained for the *PHYC* locus was ranked in this distribution. This rank gave an empirical p-value. To assess if the high mutation rate observed at the microsatellite loci [34] might impair this test, we performed a simulation study. We sampled 27 generations apart, two samples to mimic the 1976 and 2003 samples. We modelled a hundred microsatellite loci in a population presenting an effective size estimated as described in the following paragraph. We simulated microsatellite loci following a generalized stepwise model [34]–[35]. In this model, the probability of a mutation of k repeat follows a geometric distribution with a mean p [35]. This model is closer to what is generally observed in terms of microsatellite mutation [34] than a simple stepwise model. We considered a mutation rate of 10^−1^, 10^−2^, 10^−3^, 10^−4^, 10^−5^ and 10^−6^. The geometric mean p was set to 0.652 based on empirical data available on plants [34]–[35]. The average F_ST_ was calculated and plotted against the mutation rate. We also calculated the average number of alleles as it is an easier summary statistics to compare to our empirical data, since the mutation rate is generally unknown.

For the simulation, we used the two temporal samples (1976 and 2003) to calculate a population effective size N_e_ based on the 25 microsatellite loci. We used the approach of Wang [36]–[37] to obtain a likelihood point estimate of N_e_ the effective size and the distribution of the log-likelihood around this estimated effective size. We then simulated a F_ST_ distribution between two datasets sampled 27 generations apart considering only the drift effect associated with the whole population estimated effective size (N_e_). For this simulation, we used a classical Wright-Fischer model assuming a binomial law to draw individuals from one generation to the next. We considered bi-allelic loci (SNP) with a uniform frequency distribution in the initial population and simulated the frequency of the allele in the final population by drawing a binomial law to update the frequency from one generation to the next. We then simulated a dataset based on the initial population and the final population allele frequencies, and the number of individuals sampled in our real dataset in the two populations. So this simulation took both drift and sampling effect into account. We then used these two samples to calculate the differentiation F_ST_ [38]. A hundred thousand different simulations were performed, and a hundred thousand F_ST_ were calculated. We obtained a distribution of F_ST_ for bi-allelic markers. These simulations were performed by developing R code. The *PHYC* F_ST_ was then compared to the simulated distribution, and the probability of observing this particular value was estimated. We used the rank of the *PHYC* F_ST_ in the distribution and reported this rank as a p-value by dividing by the number of simulations (100000).

## Supporting Information

Figure S1 **Geographical sampling locations in Niger.** A total of 192 pearl millet accessions from 1976 and 420 accessions from 2003 were sampled in the same villages. The number of samples collected in a given village is indicated by the size of the dot.
(DOC)

Figure S2 **Morphological differences observed between cultivated and weedy morphotypes.** In this picture, two different individuals from the same accession seedlot (Pe02783) with a cultivated (left) and weedy spike morphology (right) are shown. Weedy plants are generally characterized by shorter ears, thinner stems, higher branching morphology, partial or total shattering and long bristles.
(DOC)

Figure S3 **Comparison of the number of weedy plants in the 2003 and the 1976 samples.** The number of weedy plants in each seedlot was assessed for the 2003 and 1976 samples. The figure represent the number of seedlot presenting zero, 1, 2, etc., weedy plants out of a total of 25 individuals. Two field trials were performed, one in 2004 and one in 2005.
(DOC)

Figure S4 **Principal component analysis of genotypic data.** PCA was performed on multilocus genotypes on data from the 1976 and 2003 samples. The 1976 individuals are represented by a light gray triangle and and 2003 individuals by a dark gray square. The first PCA axis explains 1.375% of the variance and the second 1.291%. The statistical difference between the two samples on the first PCA axis was not significant (Mann-Whitney test, χ^2^ = 3.62, p = 0.057) but the difference was significant on the second axis (Mann-Whitney test, χ^2^ = 5.26, p = 0.022).
(DOC)

Figure S5 **Effect of microsatellite mutation rate on F_ST._** The mean allelic F_ST_ and its standard error were calculated for two datasets sampled 27 years apart. The size of the two samples was the same as the 1976 and 2003 pearl millet samples. We modeled a population of N = 12813 individuals and a hundred microsatellite loci exhibiting a generalized stepwise mutation model. Mutation rates from 10^−1^ to 10^−6^ were simulated. The mean F_ST_ increased from 10^−1^ to 10^−3^, then from 10^−3^ to 10^−6^. F_ST_ did not vary with the mutation rate. A mutation rate of 10^−4^ corresponds to an average number of alleles of 10.8 (s.e. 2.4). The average number of alleles observed in our real dataset was 10.4.
(DOC)

Figure S6 **Population structure of the 1976 and 2003 samples.** The log likelihood for different numbers of assumed populations is given for the 1976 sample (**A**) and the 2003 sample (**B**). The ancestry for two assumed populations is given for the 1976 sample (**C**) and the 2003 sample (**D**). Even though the likelihood for K = 2 was higher in the 2003 samples, there was no clear two group ancestry structure. The structure signal detected for the 2003 sample was very weak so we considered the absence of population structure (K = 1) to be a likely hypothesis for the 2003 sample.
(DOC)

Figure S7 **Estimation of the effective size (N_e_) based on the two temporal samples.** The analysis was based on the allele frequency of the 25 different microsatellites for the two samples (1976 and 2003). The log likelihood is given for a different value of effective size. The highest log likelihood (log-L = −2156.6) was observed for Ne = 12813.
(DOC)

Table S1 **List of accessions in the 1976 and 2003 samples.**
(PDF)

Table S2 **List of varieties found in the same village in 1976 and 2003.**
(PDF)

Table S3 **Main characteristics of different varieties.**
(DOC)
