# Supplementary material for: Selection for Earlier Flowering Crop Associated with Climatic Variations in the Sahel
Source: PLoS One. 2011 May 4;6(5):e19563. doi: 10.1371/journal.pone.0019563 (PMC3087796; doi:10.1371/journal.pone.0019563)
Supplement: Figure S1 — Geographical sampling locations in Niger. A total of 192 pearl millet accessions from 1976 and 420 accessions from 2003 were sampled in the same villages. The number of samples collected in a given village is indicated by the size of the dot. (DOC) [file pone.0019563.s001.doc]

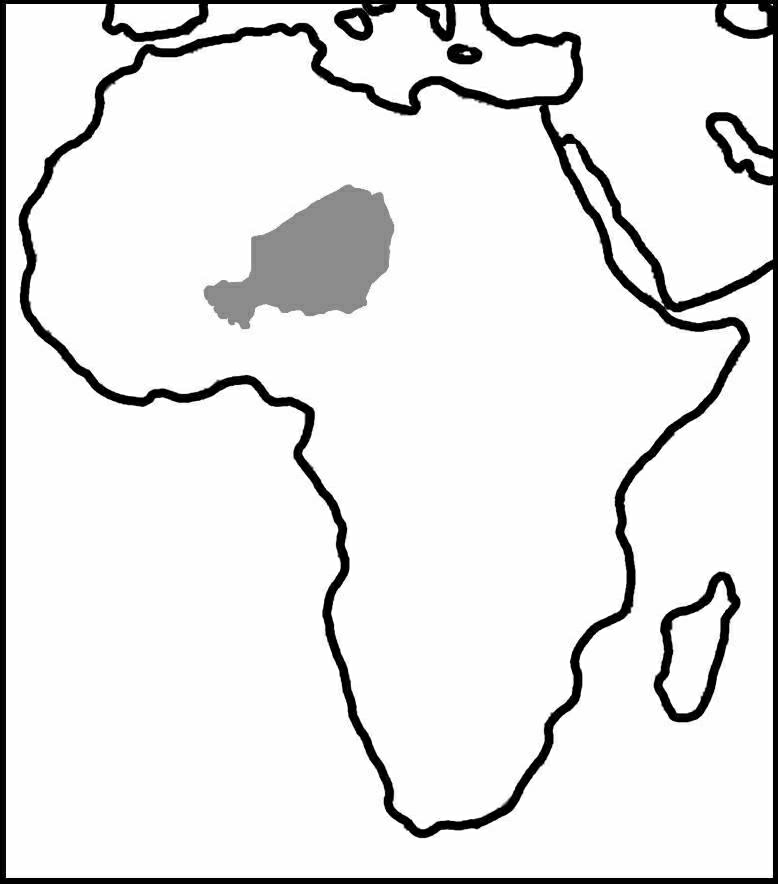
**Figure S1. Geographical sampling locations in Niger.**


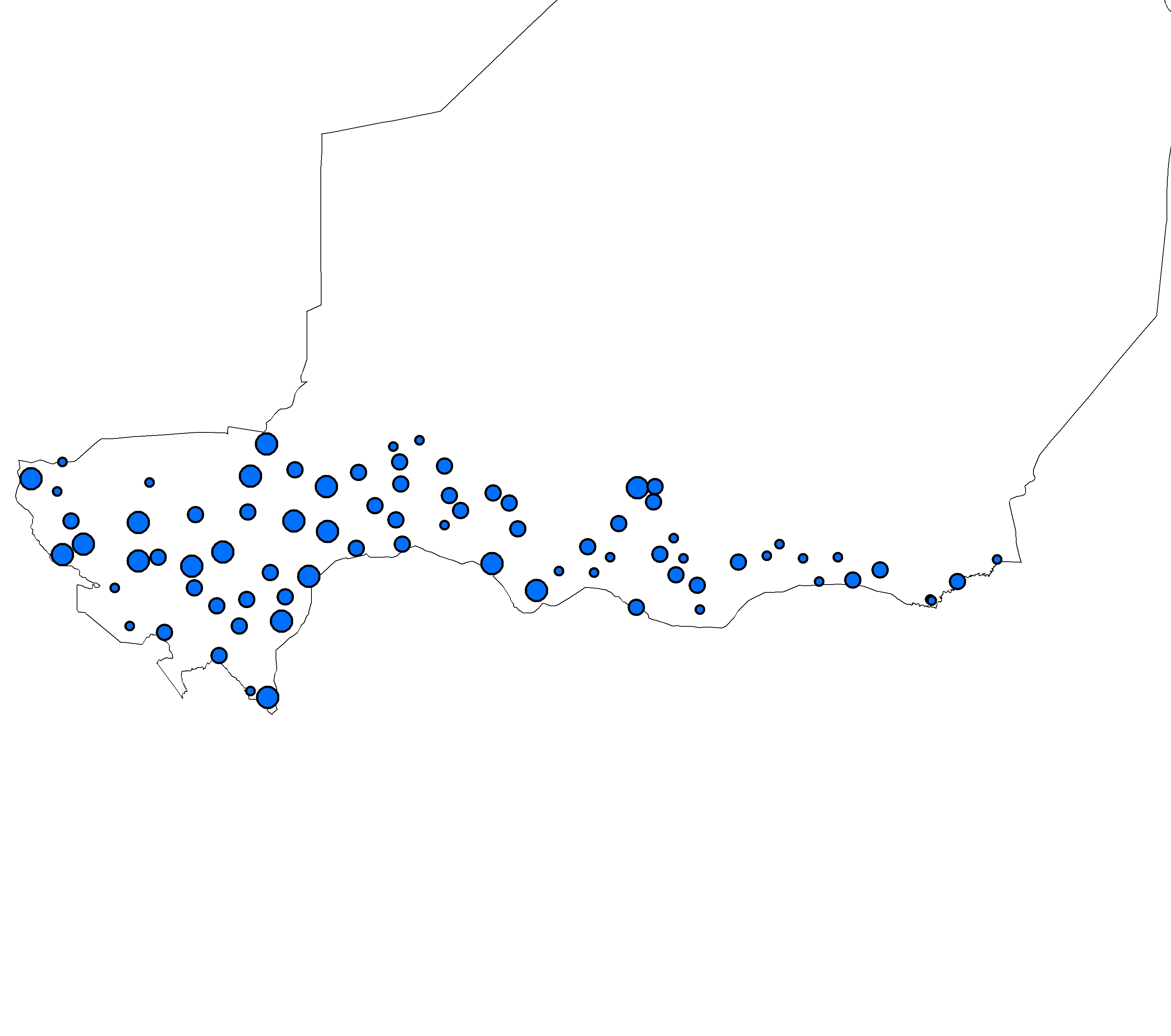

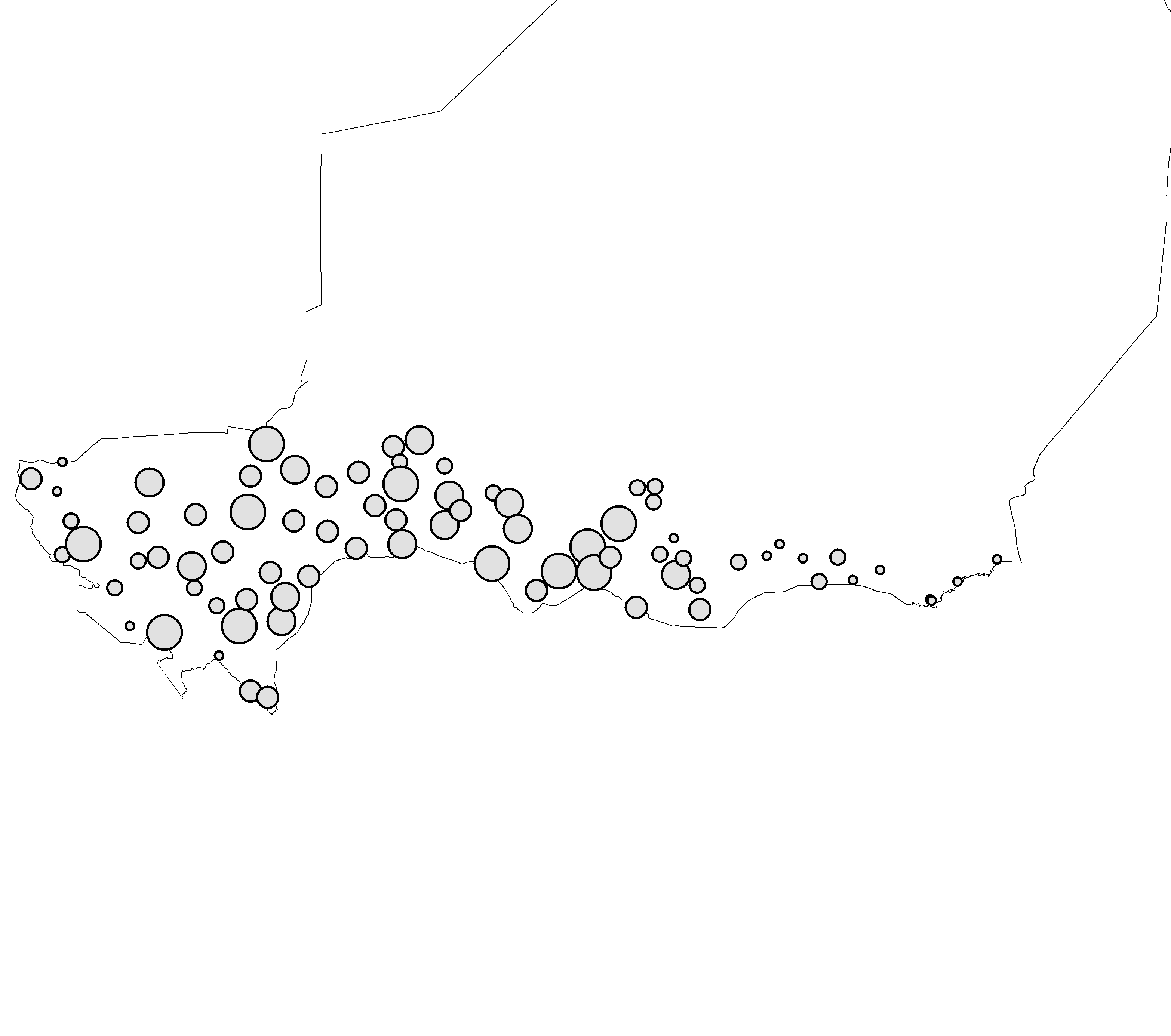


2003

1976

*Figure 1*.

In 1976, 191 Pearl millet accessions were collected in 79 villages. In 2003, 420 accessions were sampled in the same villages. The number of samples collected in a given village is represented by the size of the dot.

500km


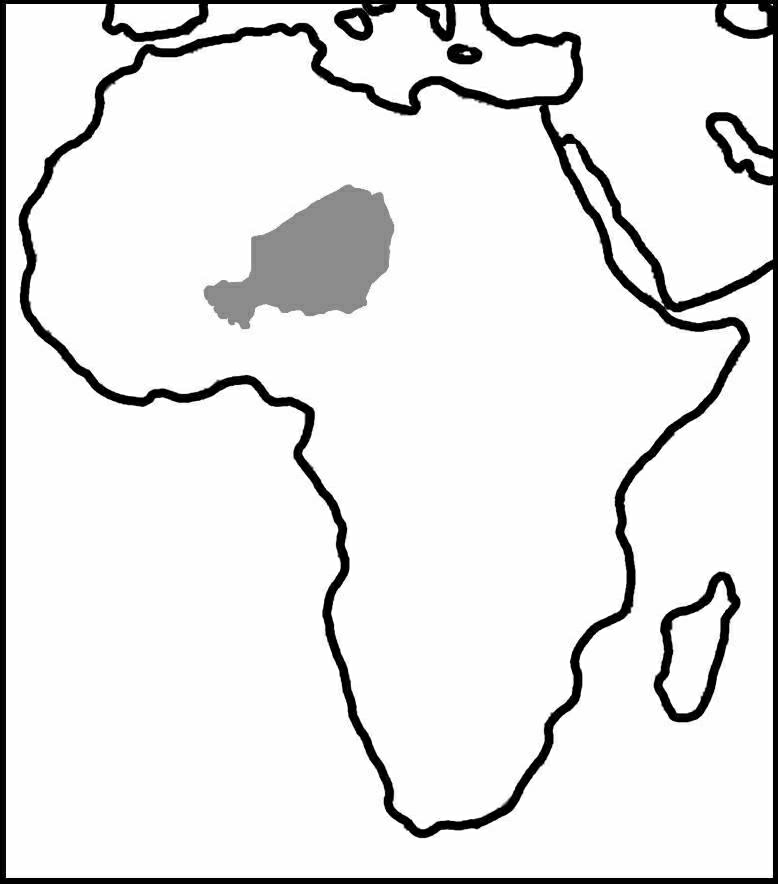

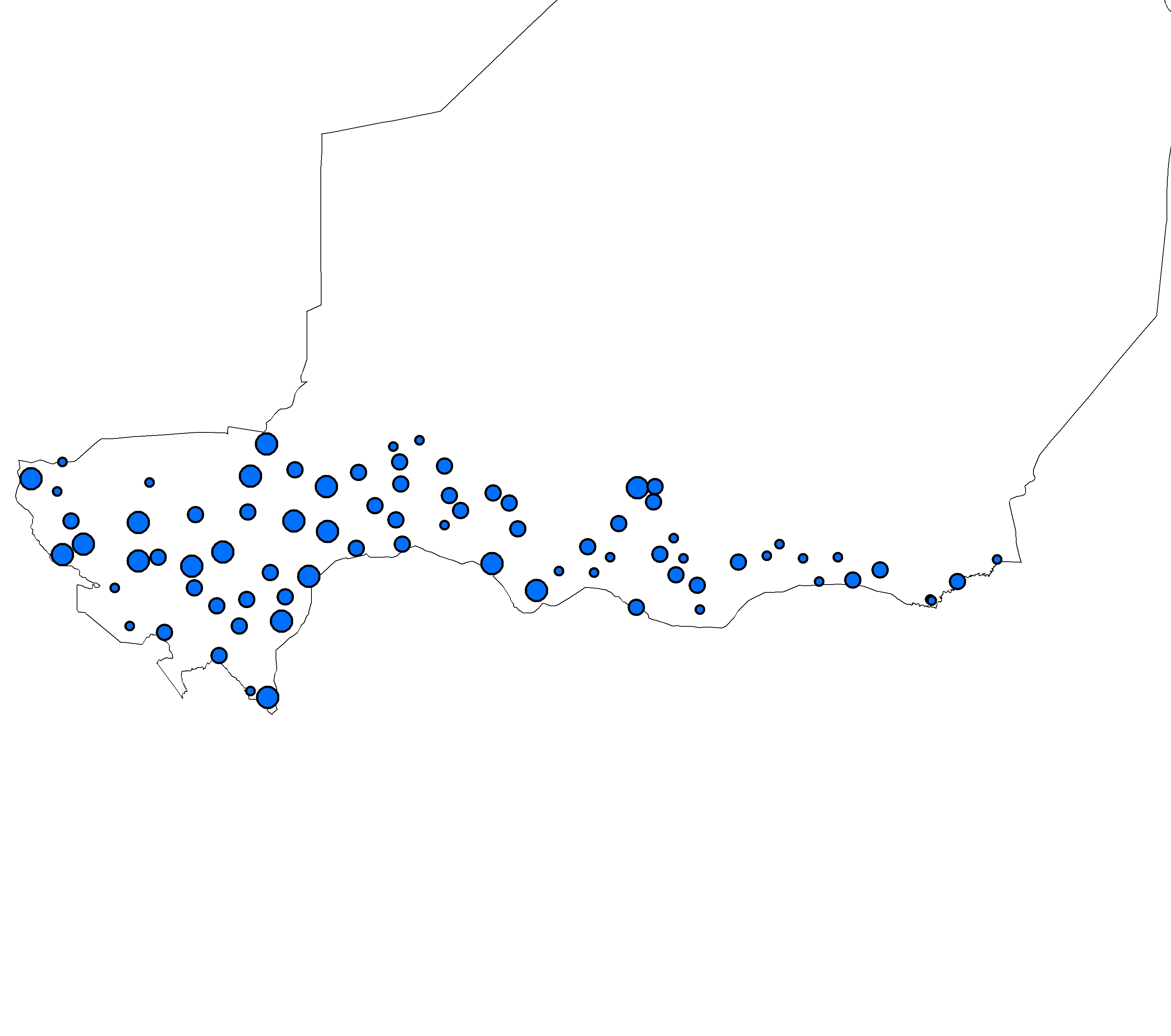

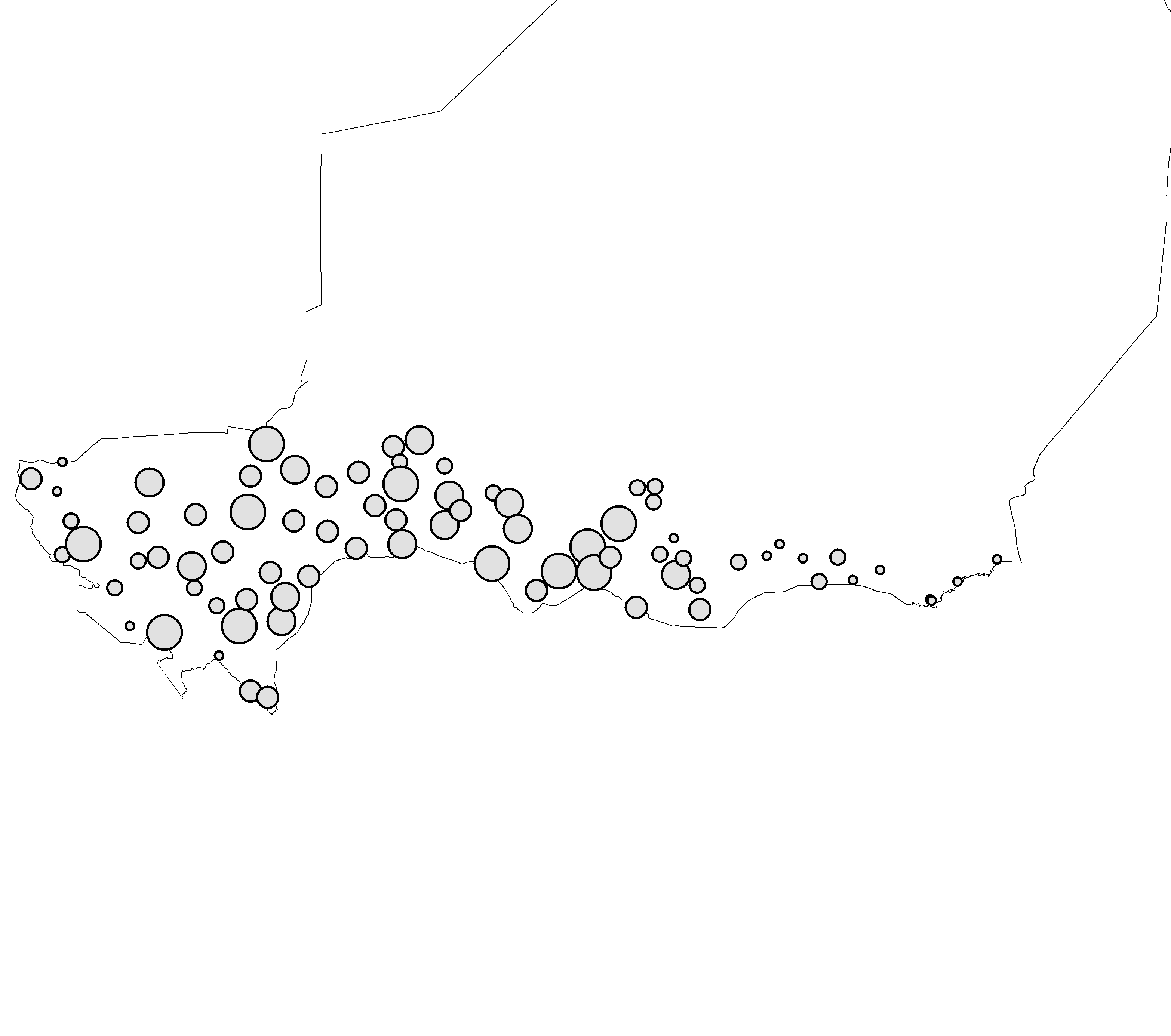


2003

1976

500km


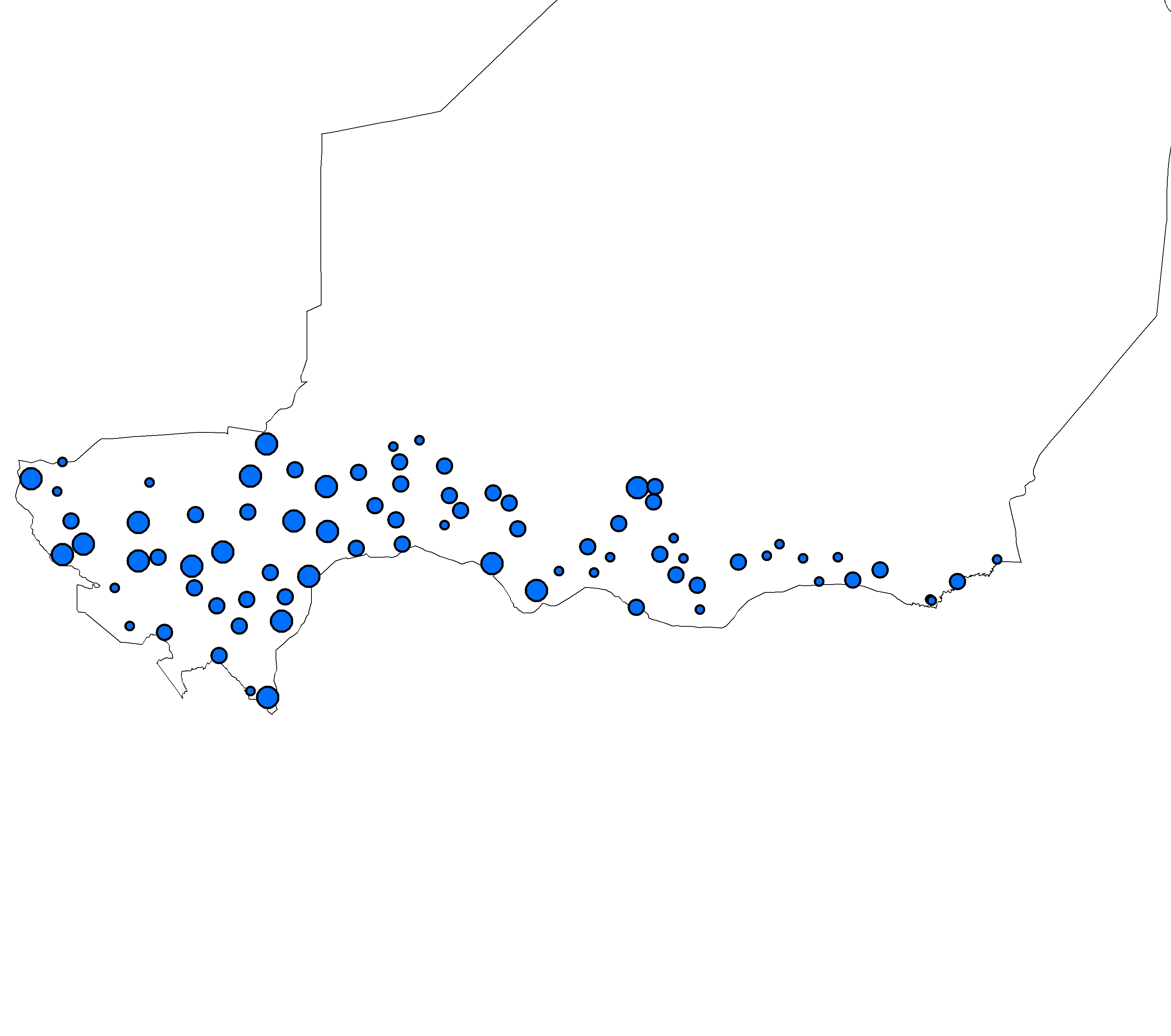

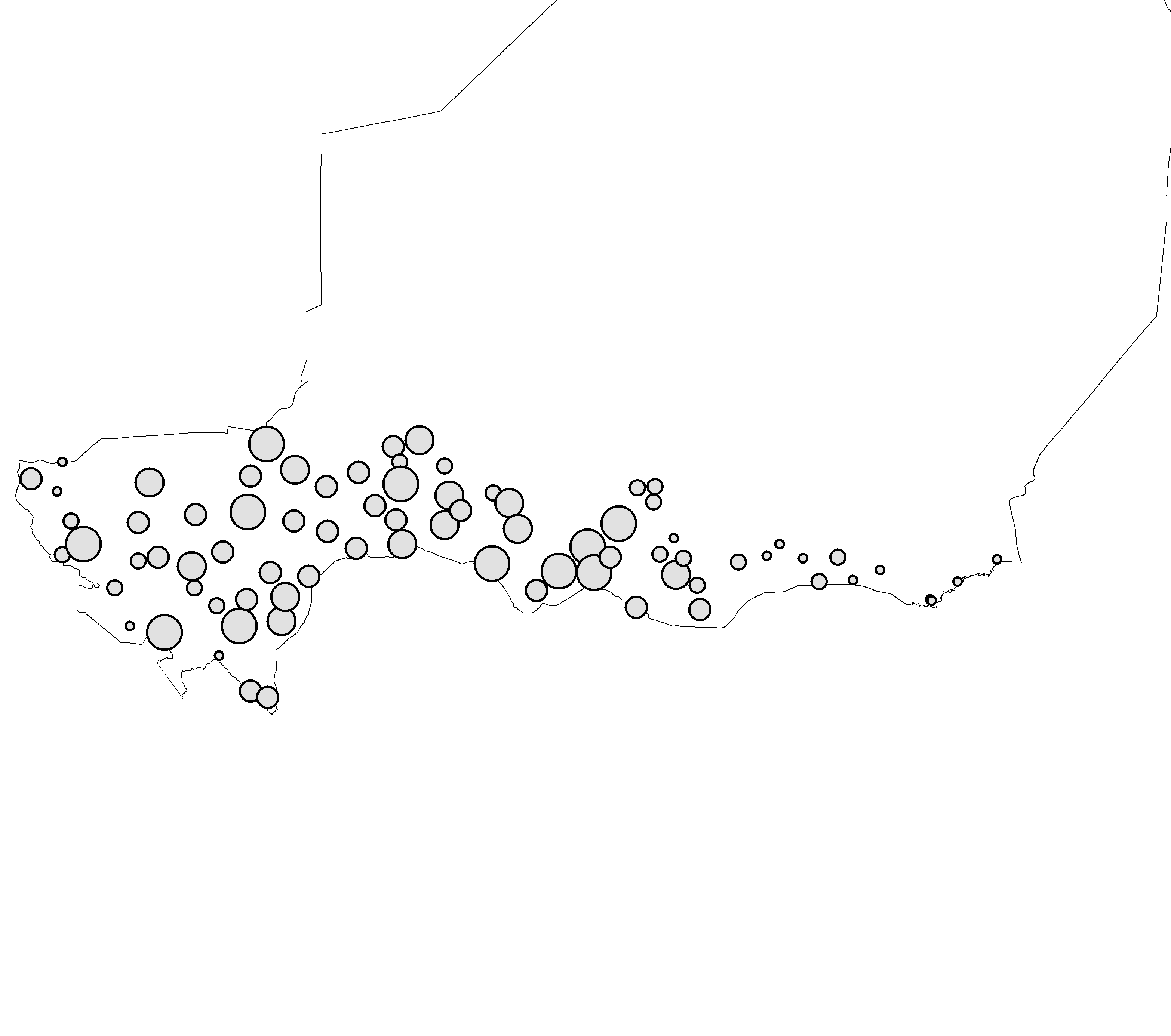


**2003**

1976


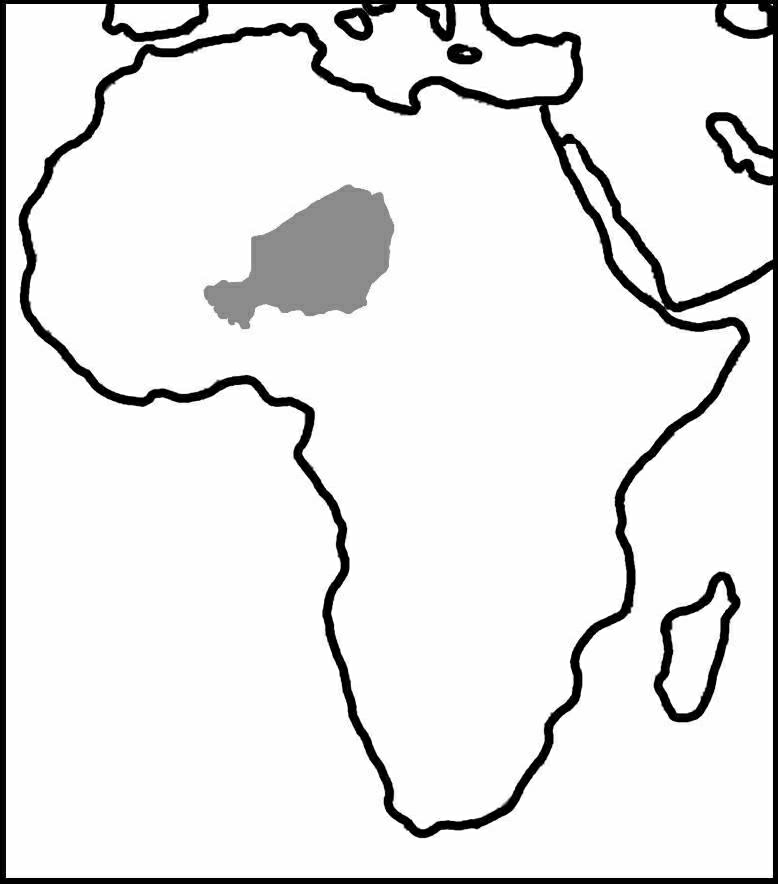


500km
