# Supplementary figures and images for: Selection for Earlier Flowering Crop Associated with Climatic Variations in the Sahel
Source: PLoS One. 2011 May 4;6(5):e19563. doi: 10.1371/journal.pone.0019563 (PMC3087796; doi:10.1371/journal.pone.0019563)

**Figure S2. Morphological differences observed between cultivated and weedy morphotypes.**

**
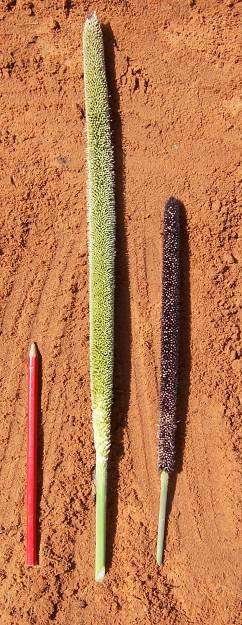
**

Supplement: Figure S2 — Morphological differences observed between cultivated and weedy morphotypes. In this picture, two different individuals from the same accession seedlot (Pe02783) with a cultivated (left) and weedy spike morphology (right) are shown. Weedy plants are generally characterized by shorter ears, thinner stems, higher branching morphology, partial or total shattering and long bristles. (DOC) [file pone.0019563.s002.doc]
