# Supplementary material for: Selection for Earlier Flowering Crop Associated with Climatic Variations in the Sahel
Source: PLoS One. 2011 May 4;6(5):e19563. doi: 10.1371/journal.pone.0019563 (PMC3087796; doi:10.1371/journal.pone.0019563)
Supplement: Figure S3 — Comparison of the number of weedy plants in the 2003 and the 1976 samples. The number of weedy plants in each seedlot was assessed for the 2003 and 1976 samples. The figure represent the number of seedlot presenting zero, 1, 2, etc., weedy plants out of a total of 25 individuals. Two field trials were performed, one in 2004 and one in 2005. (DOC) [file pone.0019563.s003.doc]

Figure S3. Comparison of the number of weedy plants in the 2003 and the 1976 samples.

2005

2003

1976

**Number of plants with weedy morphology**

**Number of plants with weedy morphology**

2004
