# Supplementary material for: Selection for Earlier Flowering Crop Associated with Climatic Variations in the Sahel
Source: PLoS One. 2011 May 4;6(5):e19563. doi: 10.1371/journal.pone.0019563 (PMC3087796; doi:10.1371/journal.pone.0019563)
Supplement: Figure S4 — Principal component analysis of genotypic data. PCA was performed on multilocus genotypes on data from the 1976 and 2003 samples. The 1976 individuals are represented by a light gray triangle and and 2003 individuals by a dark gray square. The first PCA axis explains 1.375% of the variance and the second 1.291%. The statistical difference between the two samples on the first PCA axis was not significant (Mann-Whitney test, χ2 = 3.62, p = 0.057) but the difference was significant on the second axis (Mann-Whitney test, χ2 = 5.26, p = 0.022). (DOC) [file pone.0019563.s004.doc]

**Figure S4. Principal component analysis of genotypic data.**

-2

-1

0

1

2

3

4

5

-6

-4

-2

0

2

4

2003 sample

1976 sample
