# Supplementary material for: Selection for Earlier Flowering Crop Associated with Climatic Variations in the Sahel
Source: PLoS One. 2011 May 4;6(5):e19563. doi: 10.1371/journal.pone.0019563 (PMC3087796; doi:10.1371/journal.pone.0019563)
Supplement: Figure S5 — Effect of microsatellite mutation rate on FST. The mean allelic FST and its standard error were calculated for two datasets sampled 27 years apart. The size of the two samples was the same as the 1976 and 2003 pearl millet samples. We modeled a population of N = 12813 individuals and a hundred microsatellite loci exhibiting a generalized stepwise mutation model. Mutation rates from 10−1 to 10−6 were simulated. The mean FST increased from 10−1 to 10−3, then from 10−3 to 10−6. FST did not vary with the mutation rate. A mutation rate of 10−4 corresponds to an average number of alleles of 10.8 (s.e. 2.4). The average number of alleles observed in our real dataset was 10.4. (DOC) [file pone.0019563.s005.doc]

**Figure S5. Effect of microsatellite mutation rate on F_ST._**

0

0.0001

0.0002

0.0003

0.0004

0.0005

0.0006

10^-1^

10^-2^

10^-3^

10^-4^

10^-5^

10^-6^

Mutation rate

Mean F_ST_
