# Supplementary material for: Selection for Earlier Flowering Crop Associated with Climatic Variations in the Sahel
Source: PLoS One. 2011 May 4;6(5):e19563. doi: 10.1371/journal.pone.0019563 (PMC3087796; doi:10.1371/journal.pone.0019563)
Supplement: Figure S6 — Population structure of the 1976 and 2003 samples. The log likelihood for different numbers of assumed populations is given for the 1976 sample (A) and the 2003 sample (B). The ancestry for two assumed populations is given for the 1976 sample (C) and the 2003 sample (D). Even though the likelihood for K = 2 was higher in the 2003 samples, there was no clear two group ancestry structure. The structure signal detected for the 2003 sample was very weak so we considered the absence of population structure (K = 1) to be a likely hypothesis for the 2003 sample. (DOC) [file pone.0019563.s006.doc]

**Figure S6. Population structure of the 1976 and 2003 samples**.

D.

0%

10%

20%

30%

40%

50%

60%

70%

80%

90%

100%

1

7

13

19

25

31

37

43

49

55

61

67

73

79

85

91

97

103

109

115

121

127

133

139

145

151

157

163

169

175

181

187

193

199

205

211

217

223

229

235

241

247

253

259

265

271

277

283

289

295

301

307

313

319

325

331

337

343

349

355

361

367

373

379

385

391

397

403

Individual

Ancestry

0%

10%

20%

30%

40%

50%

60%

70%

80%

90%

100%

1

7

13

19

25

31

37

43

49

55

61

67

73

79

85

91

97

103

109

115

121

127

133

139

145

151

157

163

169

175

181

187

Individual

Ancestry

C.

A.

-11500

-11300

-11100

-10900

-10700

-10500

-10300

-10100

-9900

1

2

3

4

5

**Number of populations (K)**

**LogLikelihhood**

B.

-21900

-21850

-21800

-21750

-21700

-21650

-21600

1

2

3

4

5

**Number of populations (K)**

**LogLikelihhood**
