# Supplementary material for: Selection for Earlier Flowering Crop Associated with Climatic Variations in the Sahel
Source: PLoS One. 2011 May 4;6(5):e19563. doi: 10.1371/journal.pone.0019563 (PMC3087796; doi:10.1371/journal.pone.0019563)
Supplement: Figure S7 — Estimation of the effective size (Ne) based on the two temporal samples. The analysis was based on the allele frequency of the 25 different microsatellites for the two samples (1976 and 2003). The log likelihood is given for a different value of effective size. The highest log likelihood (log-L = −2156.6) was observed for Ne = 12813. (DOC) [file pone.0019563.s007.doc]

Figure S7. Estimation of the effective size (N_e_) based on the two temporal samples

Effective size (Ne)

log-likelihood (_x_ 10^3^)

-2.159

-2.158

-2.157

-2.156

0

10000

20000

30000

40000
