# Supplementary material for: Selection for Earlier Flowering Crop Associated with Climatic Variations in the Sahel
Source: PLoS One. 2011 May 4;6(5):e19563. doi: 10.1371/journal.pone.0019563 (PMC3087796; doi:10.1371/journal.pone.0019563)
Supplement: Table S1 — List of accessions in the 1976 and 2003 samples. (PDF) [file pone.0019563.s008.pdf]

Table S1

Passport data for varieties from the 2003 and 1976 samples

For each variety, the town of origin, latitude, longitude, the year and the name of the variety are given.

Accession number	Town	Latitude	Longitude	Year of collection	Name of the variety
M371	Adoumchi	13.45N	10.14E	2003	Badendji
PE02599	Adoumchi	13.45N	10.14E	1976	Badendji
M212	Afala	15.10N	05.14E	2003	Ouianbijini
M213	Afala	15.10N	05.14E	2003	Guerguéra
M214	Afala	15.10N	05.14E	2003	Baléri
M215	Afala	15.10N	05.14E	2003	Gassama
M216	Afala	15.10N	05.14E	2003	Bakin Iri
PE02744	Afala	15.10N	05.14E	1976	Guéro
M328	Ajékoria	14.26N	06.47E	2003	P3 Kollo
M329	Ajékoria	14.26N	06.47E	2003	Guerguéra
M330	Ajékoria	14.26N	06.47E	2003	Tarnekoua
M331	Ajékoria	14.26N	06.47E	2003	Ba Angouré
M332	Ajékoria	14.26N	06.47E	2003	Zongo
M333	Ajékoria	14.26N	06.47E	2003	Ouianbijini
M334	Ajékoria	14.26N	06.47E	2003	Tarnekoua
PE02698	Ajékoria	14.26N	06.47E	1976	Zongo
PE02699	Ajékoria	14.26N	06.47E	1976	Ba Angouré
M250	Azarori	14.09N	05.55E	2003	Guerguéra
M251	Azarori	14.09N	05.55E	2003	Dan Boudouma
M252	Azarori	14.09N	05.55E	2003	Ba Angouré
M253	Azarori	14.09N	05.55E	2003	Dabro
M254	Azarori	14.09N	05.55E	2003	HKP
M255	Azarori	14.09N	05.55E	2003	Matam Hatsi
M256	Azarori	14.09N	05.55E	2003	Zatib
PE02803	Azarori	14.09N	05.55E	1976	Guerguéra
M269	Bagaroua	14.39N	04.20E	2003	Guerguéra
M270	Bagaroua	14.39N	04.20E	2003	Bakin Iri
M271	Bagaroua	14.39N	04.20E	2003	Dabki
M272	Bagaroua	14.39N	04.20E	2003	Bazaomé
M273	Bagaroua	14.39N	04.20E	2003	Eka
M274	Bagaroua	14.39N	04.20E	2003	Zongo
PE02758	Bagaroua	14.39N	04.20E	1976	Zongo
PE02759	Bagaroua	14.39N	04.20E	1976	Bakin Iri
PE02760	Bagaroua	14.39N	04.20E	1976	Guerguéra
PE02761	Bagaroua	14.39N	04.20E	1976	Bazaomé
M027	Baleyara	13.48N	02.57E	2003	Haïni Kiré

M028	Baleyara	13.48N	02.57E	2003	Tchoumo
M029	Baleyara	13.48N	02.57E	2003	Zongo
M030	Baleyara	13.48N	02.57E	2003	Haïni Kiré
M031	Baleyara	13.48N	02.57E	2003	Foulanya
M032	Baleyara	13.48N	02.57E	2003	M'Bounga
PE02895	Baleyara	13.48N	02.57E	1976	Haïni Kiré
PE02896	Baleyara	13.48N	02.57E	1976	Tchoumo
PE02897	Baleyara	13.48N	02.57E	1976	Foulanya
PE02898	Baleyara	13.48N	02.57E	1976	Somno Goberikoy
M018	Bandio	13.54N	01.05E	2003	Haïni Kiré
M019	Bandio	13.54N	01.05E	2003	Somno
M020	Bandio	13.54N	01.05E	2003	Kolala
M021	Bandio	13.54N	01.05E	2003	Gnieye Koaré
M022	Bandio	13.54N	01.05E	2003	Gnieye Kiré
M023	Bandio	13.54N	01.05E	2003	Kourmé Haïni
M024	Bandio	13.54N	01.05E	2003	Fiti-Fiti
M025	Bandio	13.54N	01.05E	2003	Gnieye -Koba
M026	Bandio	13.54N	01.05E	2003	Darankoba
PE02955	Bandio	13.54N	01.05E	1976	Gnieye
PE02956	Bandio	13.54N	01.05E	1976	Gnieye
PE02957	Bandio	13.54N	01.05E	1976	Tchoumo
PE02958	Bandio	13.54N	01.05E	1976	Kolala
M065	Banimaté	14.17N	02.35E	2003	Somno
M066	Banimaté	14.17N	02.35E	2003	Darankoba
M067	Banimaté	14.17N	02.35E	2003	Tchoumo
M068	Banimaté	14.17N	02.35E	2003	Haïni Kiré
M069	Banimaté	14.17N	02.35E	2003	Gnieye
PE02925	Banimaté	14.17N	02.35E	1976	Haïni Kiré
PE02926	Banimaté	14.17N	02.35E	1976	Somno
M007	Bankilaré	14.35N	00.44E	2003	Bondabia
M008	Bankilaré	14.35N	00.44E	2003	Enélé Wanalis
PE02969	Bankilaré	14.35N	00.44E	1976	Haïni Kiré
M077	Barakan	14.11N	01.49E	2003	Haïni Kiré
M078	Barakan	14.11N	01.49E	2003	Darankoba
M079	Barakan	14.11N	01.49E	2003	Dikabona
M080	Barakan	14.11N	01.49E	2003	Tchoumo
M081	Barakan	14.11N	01.49E	2003	Foulanya
M082	Barakan	14.11N	01.49E	2003	Somno
PE02934	Barakan	14.11N	01.49E	1976	Foulanya
PE02935	Barakan	14.11N	01.49E	1976	Tchoumo
PE02936	Barakan	14.11N	01.49E	1976	Ayoryzé

PE02937	Barakan	14.11N	01.49E	1976	Somno
PE02938	Barakan	14.11N	01.49E	1976	Haïni Kiré
M094	Bengou	11.54N	03.33E	2003	Ouianbijini
M095	Bengou	11.54N	03.33E	2003	Idon Hawaïnia
M096	Bengou	11.54N	03.33E	2003	Bakan Meawa
M097	Bengou	11.54N	03.33E	2003	Dan Tondi
M098	Bengou	11.54N	03.33E	2003	Zongo
M099	Bengou	11.54N	03.33E	2003	Maïgachi
PE02826	Bengou	11.54N	03.33E	1976	Zongo Ouianbijini
PE02827	Bengou	11.54N	03.33E	1976	Zongo
PE02828	Bengou	11.54N	03.33E	1976	Maewa
PE02829	Bengou	11.54N	03.33E	1976	Maewa Idoaouénia
M352	Bosso	13.42N	13.19E	2003	Boudouma
PE02584	Bosso	13.42N	13.19E	1976	Boudouma
M355	Boudoum	13.10N	12.15E	2003	Boudouma
M356	Boudoum	13.10N	12.15E	2003	Moro
PE02588	Boudoum	13.10N	12.15E	1976	Moro
M359	Chéri	13.26N	11.23E	2003	Fidéoua
M360	Chéri	13.26N	11.23E	2003	Moro
PE02604	Chéri	13.26N	11.23E	1976	Moro
PE02605	Chéri	13.26N	11.23E	1976	Fidéoua
M351	Chétirami	13.11N	12.25E	2003	Moro
PE02586	Chétirami	13.11N	12.25E	1976	Moro
M182	Coquièzé Koara	13.37N	02.32E	2003	Somno Bi
M183	Coquièzé Koara	13.37N	02.32E	2003	Somno Seye
M184	Coquièzé Koara	13.37N	02.32E	2003	Somno Tchangara
M185	Coquièzé Koara	13.37N	02.32E	2003	CIVT
M186	Coquièzé Koara	13.37N	02.32E	2003	Tchoumo Bi
M187	Coquièzé Koara	13.37N	02.32E	2003	Darankoba
M188	Coquièzé Koara	13.37N	02.32E	2003	Tchoumo Koaré
PE02891	Coquièzé Koara	13.37N	02.32E	1976	Tchoumo
PE02892	Coquièzé Koara	13.37N	02.32E	1976	Somno
PE02893	Coquièzé Koara	13.37N	02.32E	1976	Maewa Darankoba
PE02894	Coquièzé Koara	13.37N	02.32E	1976	Haïni Kiré Bounga
M055	Danbanguiro	14.47N	03.19E	2003	Dan Téra
M056	Danbanguiro	14.47N	03.19E	2003	Dan Hatsi
M057	Danbanguiro	14.47N	03.19E	2003	Bakin Iri
M058	Danbanguiro	14.47N	03.19E	2003	Zongo
M059	Danbanguiro	14.47N	03.19E	2003	Bazaomé
PE02921	Danbanguiro	14.47N	03.19E	1976	Bakin Iri
PE02922	Danbanguiro	14.47N	03.19E	1976	Maewa Darankoba

PE02923	Danbanguiro	14.47N	03.19E	1976	Zongo
PE02924	Danbanguiro	14.47N	03.19E	1976	Bazaomé
M378	Dengas	13.03N	09.20E	2003	Zongo
M379	Dengas	13.03N	09.20E	2003	Massangari 1
M380	Dengas	13.03N	09.20E	2003	Gamogi
M381	Dengas	13.03N	09.20E	2003	Massangari 2
M382	Dengas	13.03N	09.20E	2003	Massangari 3
PE02640	Dengas	13.03N	09.20E	1976	Ba Angouré
M237	Déoulé	14.32N	05.59E	2003	Guerguéra 1
M238	Déoulé	14.32N	05.59E	2003	Guerguéra 2
M239	Déoulé	14.32N	05.59E	2003	Kitsandamo
M240	Déoulé	14.32N	05.59E	2003	Dan Dagali
M241	Déoulé	14.32N	05.59E	2003	Bakin Hatsi
M242	Déoulé	14.32N	05.59E	2003	Bakin Hatsi 2
M243	Déoulé	14.32N	05.59E	2003	Zanfaroua
PE02800	Déoulé	14.32N	05.59E	1976	Forforon Hatsi
PE02801	Déoulé	14.32N	05.59E	1976	Guerguéra
PE02802	Déoulé	14.32N	05.59E	1976	Gassama
M389	Dogo	13.30N	09.01E	2003	Bakin Hatsi
M390	Dogo	13.30N	09.01E	2003	Gamogi
M391	Dogo	13.30N	09.01E	2003	P3 Kollo
M392	Dogo	13.30N	09.01E	2003	Zongo
M393	Dogo	13.30N	09.01E	2003	Tamangagi
M394	Dogo	13.30N	09.01E	2003	Ankoutess
M395	Dogo	13.30N	09.01E	2003	Ba Angouré
PE02646	Dogo	13.30N	09.01E	1976	Tamangagi
PE02647	Dogo	13.30N	09.01E	1976	Ba Angouré
M113	Dogon Kiria	14.04N	04.21E	2003	Jan Hatsi
M114	Dogon Kiria	14.04N	04.21E	2003	Zongo
M115	Dogon Kiria	14.04N	04.21E	2003	Bakin Iri
M116	Dogon Kiria	14.04N	04.21E	2003	Guerguéra
M117	Dogon Kiria	14.04N	04.21E	2003	Zongo
M118	Dogon Kiria	14.04N	04.21E	2003	Meawa
PE02859	Dogon Kiria	14.04N	04.21E	1976	Maewa Baki
PE02860	Dogon Kiria	14.04N	04.21E	1976	Maewa Ja
PE02861	Dogon Kiria	14.04N	04.21E	1976	Guerguéra
PE02862	Dogon Kiria	14.04N	04.21E	1976	Bakin Iri
PE02863	Dogon Kiria	14.04N	04.21E	1976	Dabki
PE02864	Dogon Kiria	14.04N	04.21E	1976	Zongo Outchabiri
M217	Edir	14.50N	04.46E	2003	Guerguéra 1
M218	Edir	14.50N	04.46E	2003	Guerguéra 2

M219	Edir	14.50N	04.46E	2003	Zongo
M220	Edir	14.50N	04.46E	2003	Ouianbijini
M221	Edir	14.50N	04.46E	2003	HKP
M222	Edir	14.50N	04.46E	2003	Bakin Iri
PE02754	Edir	14.50N	04.46E	1976	Zongo
PE02755	Edir	14.50N	04.46E	1976	Guerguéra
M083	Eléwayene	14.58N	00.48E	2003	Enélé Wazagarane
PE02943	Eléwayene	14.58N	00.48E	1976	Haïni Kiré
M128	Falwel	13.32N	03.35E	2003	Zongo
M129	Falwel	13.32N	03.35E	2003	Somno Gaoré
M130	Falwel	13.32N	03.35E	2003	Eka
M131	Falwel	13.32N	03.35E	2003	Somno Bi
M132	Falwel	13.32N	03.35E	2003	Haïni Kiré
PE02885	Falwel	13.32N	03.35E	1976	Somno Gaoré
PE02886	Falwel	13.32N	03.35E	1976	Haïni Kiré
PE02887	Falwel	13.32N	03.35E	1976	Somno Bi
M404	Gangara	14.38N	08.30E	2003	Tamangagi
M405	Gangara	14.38N	08.30E	2003	Ankoutess Filé
M406	Gangara	14.38N	08.30E	2003	Ankoutess Béra
M407	Gangara	14.38N	08.30E	2003	Ba Angouré
PE02663	Gangara	14.38N	08.30E	1976	Ankoutess
PE02664	Gangara	14.38N	08.30E	1976	Tamangagi
PE02665	Gangara	14.38N	08.30E	1976	Filé
PE02666	Gangara	14.38N	08.30E	1976	Ba Angouré
M289	Gazaoua	13.32N	07.55E	2003	Zanfaroua
M290	Gazaoua	13.32N	07.55E	2003	Zongo
M291	Gazaoua	13.32N	07.55E	2003	Ankoutess
M292	Gazaoua	13.32N	07.55E	2003	Ankoutess
M293	Gazaoua	13.32N	07.55E	2003	Dan Moussa
M294	Gazaoua	13.32N	07.55E	2003	P3 Kollo
M295	Gazaoua	13.32N	07.55E	2003	Dan Eka
M296	Gazaoua	13.32N	07.55E	2003	CIVT
M297	Gazaoua	13.32N	07.55E	2003	HKP
M298	Gazaoua	13.32N	07.55E	2003	Ouianbijini
M299	Gazaoua	13.32N	07.55E	2003	Ankoutess
M300	Gazaoua	13.32N	07.55E	2003	Dan Tiama
M301	Gazaoua	13.32N	07.55E	2003	Dan Gombé
M302	Gazaoua	13.32N	07.55E	2003	Bakan Maewa
PE02717	Gazaoua	13.32N	07.55E	1976	Zongo
M365	Goudoumaria	13.44N	11.11E	2003	Fidéoua
M366	Goudoumaria	13.44N	11.11E	2003	Badendji+Gassia

PE02594	Goudoumaria	13.44N	11.11E	1976	Moro
M408	Guézawa	14.27N	08.43E	2003	Ba Angouré
M409	Guézawa	14.27N	08.43E	2003	Ankoutess
M410	Guézawa	14.27N	08.43E	2003	Dan Eka
M411	Guézawa	14.27N	08.43E	2003	Tamangagi
PE02658	Guézawa	14.27N	08.43E	1976	Ba Angouré
PE02659	Guézawa	14.27N	08.43E	1976	Ankoutess
M372	Guidiguir	13.40N	09.51E	2003	Ba Angouré
M373	Guidiguir	13.40N	09.51E	2003	Badendji
M374	Guidiguir	13.40N	09.51E	2003	Koudous
PE02629	Guidiguir	13.40N	09.51E	1976	Bodendji
PE02630	Guidiguir	13.40N	09.51E	1976	Goudis
M233	Ibohamane	14.55N	05.55E	2003	Dan Barnou
M234	Ibohamane	14.55N	05.55E	2003	Zongo
M235	Ibohamane	14.55N	05.55E	2003	Guerguéra
M236	Ibohamane	14.55N	05.55E	2003	Ejeg
PE02790	Ibohamane	14.55N	05.55E	1976	Guerguéra
PE02791	Ibohamane	14.55N	05.55E	1976	Ejeg
M263	Jinguines	14.24N	04.59E	2003	Meawa
M264	Jinguines	14.24N	04.59E	2003	Bakin Iri
M265	Jinguines	14.24N	04.59E	2003	Guéro
M266	Jinguines	14.24N	04.59E	2003	Guerguéra
M267	Jinguines	14.24N	04.59E	2003	Dogon Iri
M268	Jinguines	14.24N	04.59E	2003	HKP
PE02763	Jinguines	14.24N	04.59E	1976	Guéro
PE02764	Jinguines	14.24N	04.59E	1976	Guerguéra
PE02765	Jinguines	14.24N	04.59E	1976	Bakin Iri
M312	Kanan Bakaché	13.52N	07.50E	2003	Dan Tiama-Niger
M313	Kanan Bakaché	13.52N	07.50E	2003	Dan Tiama-Nigeria
M314	Kanan Bakaché	13.52N	07.50E	2003	Ankoutess Hélé
M315	Kanan Bakaché	13.52N	07.50E	2003	Zongo
M316	Kanan Bakaché	13.52N	07.50E	2003	Dan Tiama 3
M317	Kanan Bakaché	13.52N	07.50E	2003	Ouianbijini
M318	Kanan Bakaché	13.52N	07.50E	2003	Zongo
M319	Kanan Bakaché	13.52N	07.50E	2003	Zanfaroua
M320	Kanan Bakaché	13.52N	07.50E	2003	Tamangagi 1
M321	Kanan Bakaché	13.52N	07.50E	2003	Tamangagi 2
M322	Kanan Bakaché	13.52N	07.50E	2003	Ba Angouré
M323	Kanan Bakaché	13.52N	07.50E	2003	Danguéguimi
PE02709	Kanan Bakaché	13.52N	07.50E	1976	Zongo
PE02710	Kanan Bakaché	13.52N	07.50E	1976	Dan Tiama

PE02711	Kanan Bakaché	13.52N	07.50E	1976	Ankoutess
M179	Karma	13.41N	01.49E	2003	Tchoumo
M180	Karma	13.41N	01.49E	2003	Haïni Kiré
M181	Karma	13.41N	01.49E	2003	Décigandey
PE02949	Karma	13.41N	01.49E	1976	Haïni Kiré
PE02950	Karma	13.41N	01.49E	1976	Maewa Darankoba
PE02951	Karma	13.41N	01.49E	1976	Tchoumo
PE02952	Karma	13.41N	01.49E	1976	Somno Koiré
M244	Karofane	14.19N	06.08E	2003	Zongo 1
M245	Karofane	14.19N	06.08E	2003	HKP
M246	Karofane	14.19N	06.08E	2003	Zongo 2
M247	Karofane	14.19N	06.08E	2003	Guerguéa
M248	Karofane	14.19N	06.08E	2003	Bakin Iri
M249	Karofane	14.19N	06.08E	2003	Ba Angouré
PE02797	Karofane	14.19N	06.08E	1976	Guerguéra
PE02798	Karofane	14.19N	06.08E	1976	Ba Angouré
PE02799	Karofane	14.19N	06.08E	1976	Zongo
M357	Kélakam	13.34N	11.45E	2003	Koudous
M358	Kélakam	13.34N	11.45E	2003	Moro
PE02591	Kélakam	13.34N	11.45E	1976	Moro
PE02593	Kélakam	13.34N	11.45E	1976	Boudouma
M108	Kiessé	13.29N	04.06E	2003	Zongo Kollo
M109	Kiessé	13.29N	04.06E	2003	Guerguéra
M110	Kiessé	13.29N	04.06E	2003	Bakin Iri
M111	Kiessé	13.29N	04.06E	2003	Zongo
M112	Kiessé	13.29N	04.06E	2003	Bazaomé
PE02847	Kiessé	13.29N	04.06E	1976	Zongo
PE02848	Kiessé	13.29N	04.06E	1976	Matam Hatsi
PE02849	Kiessé	13.29N	04.06E	1976	Bakin Iri
PE02850	Kiessé	13.29N	04.06E	1976	Bazaomé
PE02851	Kiessé	13.29N	04.06E	1976	Maewa Bari
PE02852	Kiessé	13.29N	04.06E	1976	Guerguéra
M145	Kikoudou Koara	12.50N	03.10E	2003	Somno Gaoré Seye
M146	Kikoudou Koara	12.50N	03.10E	2003	Somno Gaoré Tchangaré
M147	Kikoudou Koara	12.50N	03.10E	2003	Somno Bi Koukou
M148	Kikoudou Koara	12.50N	03.10E	2003	Somno Gao Doungourio
M149	Kikoudou Koara	12.50N	03.10E	2003	Nihinnéno
M150	Kikoudou Koara	12.50N	03.10E	2003	Eka
M151	Kikoudou Koara	12.50N	03.10E	2003	Haïni Kiré M'Bounga
M152	Kikoudou Koara	12.50N	03.10E	2003	Sambéra Izé
M153	Kikoudou Koara	12.50N	03.10E	2003	Somno Bi Warga

M154	Kikoudou Koara	12.50N	03.10E	2003	Somno Gaoré Koukou
PE02888	Kikoudou Koara	12.50N	03.10E	1976	Haïni Kiré Bounga
PE02889	Kikoudou Koara	12.50N	03.10E	1976	Somno
M368	Kilakina	13.43N	10.43E	2003	Fidéoua
M369	Kilakina	13.43N	10.43E	2003	Ba Angouré
PE02597	Kilakina	13.43N	10.43E	1976	Ba Angouré
M361	Kojiméri	13.25N	10.56E	2003	Boudouma
M362	Kojiméri	13.25N	10.56E	2003	Badendji
M363	Kojiméri	13.25N	10.56E	2003	Moro
M364	Kojiméri	13.25N	10.56E	2003	Fidéoua
PE02603	Kojiméri	13.25N	10.56E	1976	Moro
M009	Kokoro	14.12N	00.55E	2003	Katta Haïni
M010	Kokoro	14.12N	00.55E	2003	Bondabia
M011	Kokoro	14.12N	00.55E	2003	Tchinguel
M012	Kokoro	14.12N	00.55E	2003	Kolala
PE02966	Kokoro	14.12N	00.55E	1976	Tchinguel
PE02967	Kokoro	14.12N	00.55E	1976	Gnieye
PE02968	Kokoro	14.12N	00.55E	1976	Kolala
M230	Koloma Baba	14.58N	05.19E	2003	Gassama
M231	Koloma Baba	14.58N	05.19E	2003	Guerguéra
M232	Koloma Baba	14.58N	05.19E	2003	HKP
PE02785	Koloma Baba	14.58N	05.19E	1976	Guerguéra
PE02786	Koloma Baba	14.58N	05.19E	1976	Gassama
M169	Koné Béri	13.44N	02.05E	2003	Tchoumo
M170	Koné Béri	13.44N	02.05E	2003	Darankoba
M171	Koné Béri	13.44N	02.05E	2003	Dikabona
M172	Koné Béri	13.44N	02.05E	2003	Somno Koaré
M173	Koné Béri	13.44N	02.05E	2003	Haïni Kiré
PE02946	Koné Béri	13.44N	02.05E	1976	Maewa Darankoba
PE02947	Koné Béri	13.44N	02.05E	1976	Tchoumo
M335	Kornaka	14.06N	06.54E	2003	P3 Kollo
M336	Kornaka	14.06N	06.54E	2003	HKP
M337	Kornaka	14.06N	06.54E	2003	Zanfaroua
M338	Kornaka	14.06N	06.54E	2003	Guerguéra
M339	Kornaka	14.06N	06.54E	2003	Ouianbijini
M340	Kornaka	14.06N	06.54E	2003	Zongo
M341	Kornaka	14.06N	06.54E	2003	Ba Angouré
PE02696	Kornaka	14.06N	06.54E	1976	Ba Angouré
PE02697	Kornaka	14.06N	06.54E	1976	Zongo
M324	Korohane	14.34N	06.34E	2003	Guerguéra
M325	Korohane	14.34N	06.34E	2003	Ba Angouré

M326	Korohane	14.34N	06.34E	2003	Tarnakoua
M327	Korohane	14.34N	06.34E	2003	Matam Hatsi
PE02795	Korohane	14.34N	06.34E	1976	Guerguéra
PE02796	Korohane	14.34N	06.34E	1976	Ba Angouré
M087	Kotaki	12.27N	02.54E	2003	Haïni Kiré Nassounga
M088	Kotaki	12.27N	02.54E	2003	Somno
PE02820	Kotaki	12.27N	02.54E	1976	Haïni Kiré Nassounga
PE02821	Kotaki	12.27N	02.54E	1976	Haïni Kiré
PE02822	Kotaki	12.27N	02.54E	1976	Somno
M100	Lido	12.54N	03.44E	2003	Zongo
M101	Lido	12.54N	03.44E	2003	Darankoba 1
M102	Lido	12.54N	03.44E	2003	Bazaomé
M103	Lido	12.54N	03.44E	2003	Dan Eka 1
M104	Lido	12.54N	03.44E	2003	Jan Guéro
M105	Lido	12.54N	03.44E	2003	Dan Eka 2
M106	Lido	12.54N	03.44E	2003	Darankoba 2
M107	Lido	12.54N	03.44E	2003	Bakan Meawa
PE02836	Lido	12.54N	03.44E	1976	Guéro
PE02837	Lido	12.54N	03.44E	1976	Maewa
PE02838	Lido	12.54N	03.44E	1976	Darankoba
PE02839	Lido	12.54N	03.44E	1976	Zongo
PE02840	Lido	12.54N	03.44E	1976	Bazaomé
M133	Madalaïzé	13.13N	03.47E	2003	Somno Gaoré
M134	Madalaïzé	13.13N	03.47E	2003	Zongo
M135	Madalaïzé	13.13N	03.47E	2003	Eka Maryo
M136	Madalaïzé	13.13N	03.47E	2003	Somno Bi
M137	Madalaïzé	13.13N	03.47E	2003	Eka Seyo
M138	Madalaïzé	13.13N	03.47E	2003	Bazaomé
M139	Madalaïzé	13.13N	03.47E	2003	Eka Warga
PE02879	Madalaïzé	13.13N	03.47E	1976	Haïni Kiré
PE02880	Madalaïzé	13.13N	03.47E	1976	Somno Bi
PE02881	Madalaïzé	13.13N	03.47E	1976	Somno Koïré
M275	Madarounfa	13.18N	07.09E	2003	Dan Kazagui
M276	Madarounfa	13.18N	07.09E	2003	Ouianbijini
M277	Madarounfa	13.18N	07.09E	2003	Zongo
M278	Madarounfa	13.18N	07.09E	2003	Meawa
M279	Madarounfa	13.18N	07.09E	2003	Zanfaroua
PE02683	Madarounfa	13.18N	07.09E	1976	Zongo
PE02684	Madarounfa	13.18N	07.09E	1976	Zanfaroua
PE02685	Madarounfa	13.18N	07.09E	1976	Dan Kasaguey
PE02686	Madarounfa	13.18N	07.09E	1976	Dan Gambé

PE02687	Madarounfa	13.18N	07.09E	1976	Maewa
M174	Makalondi	12.50N	01.42E	2003	Duyé
M175	Makalondi	12.50N	01.42E	2003	Pagnimintay
PE02981	Makalondi	12.50N	01.42E	1976	Somno
M070	Mangaïzé	14.42N	01.58E	2003	Tchoumo
M071	Mangaïzé	14.42N	01.58E	2003	Haïni Koaré
M072	Mangaïzé	14.42N	01.58E	2003	Haïni Kiré
M073	Mangaïzé	14.42N	01.58E	2003	Ayoryzé 1
M074	Mangaïzé	14.42N	01.58E	2003	Koundasso
M075	Mangaïzé	14.42N	01.58E	2003	Ayoryzé 2
M076	Mangaïzé	14.42N	01.58E	2003	Darankoba
PE02933	Mangaïzé	14.42N	01.58E	1976	Haïni Kiré
M084	Margou	13.06N	02.52E	2003	Haïni Kiré
M085	Margou	13.06N	02.52E	2003	Haïni Kiré
M086	Margou	13.06N	02.52E	2003	Somno
PE02816	Margou	13.06N	02.52E	1976	Haïni Kiré
PE02817	Margou	13.06N	02.52E	1976	Somno
M303	May Guizawa	14.10N	08.15E	2003	Dan Zagaro
M304	May Guizawa	14.10N	08.15E	2003	Tarnakoua
M305	May Guizawa	14.10N	08.15E	2003	Dan Tiama
M306	May Guizawa	14.10N	08.15E	2003	Ankoutess
M307	May Guizawa	14.10N	08.15E	2003	Dan Kollo
M308	May Guizawa	14.10N	08.15E	2003	Zongo
M309	May Guizawa	14.10N	08.15E	2003	Ouianbijini
M310	May Guizawa	14.10N	08.15E	2003	Ankoutess Hélé
M311	May Guizawa	14.10N	08.15E	2003	Ba Angouré
PE02712	May Guizawa	14.10N	08.15E	1976	Zongo
PE02713	May Guizawa	14.10N	08.15E	1976	Ankoutess
M420	May Jirgui	13.44N	08.08E	2003	Zongo
M421	May Jirgui	13.44N	08.08E	2003	Dan Tiama
M422	May Jirgui	13.44N	08.08E	2003	Ankoutess
M423	May Jirgui	13.44N	08.08E	2003	Dan Digali
M424	May Jirgui	13.44N	08.08E	2003	Matam Hatsi
PE02715	May Jirgui	13.44N	08.08E	1976	Zongo
M396	Miria	13.43N	09.07E	2003	Ba Angouré
M397	Miria	13.43N	09.07E	2003	Gamogi
M398	Miria	13.43N	09.07E	2003	Ankoutess
M399	Miria	13.43N	09.07E	2003	Dan Tiama
PE02628	Miria	13.43N	09.07E	1976	Gamogi
M203	Mogueur	14.41N	05.20E	2003	HKP
M204	Mogueur	14.41N	05.20E	2003	Dan Boukoutou

M205	Mogueur	14.41N	05.20E	2003	Damagagari 1
M206	Mogueur	14.41N	05.20E	2003	Damagagari 2
M207	Mogueur	14.41N	05.20E	2003	Ouianbijini
M208	Mogueur	14.41N	05.20E	2003	Guerguéra
M209	Mogueur	14.41N	05.20E	2003	Zongo
M210	Mogueur	14.41N	05.20E	2003	Bakin Iri
M211	Mogueur	14.41N	05.20E	2003	Jan Guerguéra
PE02783	Mogueur	14.41N	05.20E	1976	Guerguéra
PE02784	Mogueur	14.41N	05.20E	1976	Dogon Iri
M140	Moko	13.11N	03.16E	2003	Zongo
M141	Moko	13.11N	03.16E	2003	Somno Gaoré
M142	Moko	13.11N	03.16E	2003	Somno Bi
M143	Moko	13.11N	03.16E	2003	Nihinnéno
M144	Moko	13.11N	03.16E	2003	Haïni Kiré Zéna
PE02809	Moko	13.11N	03.16E	1976	Somno
PE02810	Moko	13.11N	03.16E	1976	Haïni Kiré
M353	N'Galoua	13.25N	12.47E	2003	Boudouma
M354	N'Galoua	13.25N	12.47E	2003	Guissiri
PE02582	N'Galoua	13.25N	12.47E	1976	Boudouma
PE02583	N'Galoua	13.25N	12.47E	1976	Guissiri
M375	Ouacha	13.22N	09.18E	2003	Dan Tiama
M376	Ouacha	13.22N	09.18E	2003	Batou Kouché
M377	Ouacha	13.22N	09.18E	2003	Gamogi
PE02624	Ouacha	13.22N	09.18E	1976	Massangari
PE02625	Ouacha	13.22N	09.18E	1976	Gamogi
M342	Rwafin Wada	13.39N	06.33E	2003	Matam Hatsi
M343	Rwafin Wada	13.39N	06.33E	2003	Dan Maewa
M344	Rwafin Wada	13.39N	06.33E	2003	Zongo
M345	Rwafin Wada	13.39N	06.33E	2003	Gwacki
M346	Rwafin Wada	13.39N	06.33E	2003	Damro
M347	Rwafin Wada	13.39N	06.33E	2003	Ba Angouré
M348	Rwafin Wada	13.39N	06.33E	2003	Ouianbijini
M349	Rwafin Wada	13.39N	06.33E	2003	Guerguéra
M350	Rwafin Wada	13.39N	06.33E	2003	Zanfaroua
PE02722	Rwafin Wada	13.39N	06.33E	1976	Zongo
PE02723	Rwafin Wada	13.39N	06.33E	1976	Matam Hatsi
PE02724	Rwafin Wada	13.39N	06.33E	1976	Maewa Fola
PE02725	Rwafin Wada	13.39N	06.33E	1976	Maewa Lambara
M400	Sabon Kafi	14.39N	08.44E	2003	Ankoutess
M401	Sabon Kafi	14.39N	08.44E	2003	Ankoutess
M402	Sabon Kafi	14.39N	08.44E	2003	Ba Angouré

M403	Sabon Kafi	14.39N	08.44E	2003	Ankoutess Hélé
PE02660	Sabon Kafi	14.39N	08.44E	1976	Ankoutess
PE02661	Sabon Kafi	14.39N	08.44E	1976	Ba Angouré
M412	Sabongari	13.59N	08.59E	2003	Ba Angouré
M412+	Sabongari	13.59N	08.59E	2003	Ba Angouré
M413	Sabongari	13.59N	08.59E	2003	Tamangagi
M414	Sabongari	13.59N	08.59E	2003	Ankoutess
M415	Sabongari	13.59N	08.59E	2003	P3 Kollo
PE02653	Sabongari	13.59N	08.59E	1976	Ba Angouré
PE02654	Sabongari	13.59N	08.59E	1976	Goundou Goundou
M037	Sanam	14.52N	03.55E	2003	Guerguéra
M038	Sanam	14.52N	03.55E	2003	Dan Ayorou
M039	Sanam	14.52N	03.55E	2003	Zongo
M040	Sanam	14.52N	03.55E	2003	Bakin Iri
M041	Sanam	14.52N	03.55E	2003	Bazaomé
M042	Sanam	14.52N	03.55E	2003	Eka
M043	Sanam	14.52N	03.55E	2003	Maewa
PE02908	Sanam	14.52N	03.55E	1976	Zongo
PE02909	Sanam	14.52N	03.55E	1976	Bazaomé
PE02910	Sanam	14.52N	03.55E	1976	Guerguéra
M176	Saoura	13.20N	01.30E	2003	Somno
M177	Saoura	13.20N	01.30E	2003	Bondabi Haïni Bi
M178	Saoura	13.20N	01.30E	2003	Darankoba
PE02979	Saoura	13.20N	01.30E	1976	Somno
M370	Sisya	13.54N	10.24E	2003	Badendji
PE02598	Sisya	13.54N	10.24E	1976	Bodendji
M122	Soukoukoutan	14.12N	03.54E	2003	Bakin Iri
M123	Soukoukoutan	14.12N	03.54E	2003	Guerguéra 1
M124	Soukoukoutan	14.12N	03.54E	2003	Zongo
M125	Soukoukoutan	14.12N	03.54E	2003	Bazaomé
M126	Soukoukoutan	14.12N	03.54E	2003	Guerguéra 2
M127	Soukoukoutan	14.12N	03.54E	2003	Maewa+Guerguéra
PE02868	Soukoukoutan	14.12N	03.54E	1976	Zongo
PE02869	Soukoukoutan	14.12N	03.54E	1976	Bakin Iri
PE02870	Soukoukoutan	14.12N	03.54E	1976	Guerguéra
PE02871	Soukoukoutan	14.12N	03.54E	1976	Maewa Baki
PE02872	Soukoukoutan	14.12N	03.54E	1976	Bazaomé
PE02873	Soukoukoutan	14.12N	03.54E	1976	Maewa Fara
M223	Tabalak	15.15N	05.35E	2003	Ouitchiabiri
M224	Tabalak	15.15N	05.35E	2003	Ouianbijini
M225	Tabalak	15.15N	05.35E	2003	Guerguéra 1

M226	Tabalak	15.15N	05.35E	2003	Guerguéra 2
M227	Tabalak	15.15N	05.35E	2003	Zongo Zoungou
M228	Tabalak	15.15N	05.35E	2003	Zongo
M229	Tabalak	15.15N	05.35E	2003	Ankoutess
PE02748	Tabalak	15.15N	05.35E	1976	Guerguéra
M257	Tajaé	14.13N	05.16E	2003	Guerguéra
M258	Tajaé	14.13N	05.16E	2003	Dabki
M259	Tajaé	14.13N	05.16E	2003	Gassama
M260	Tajaé	14.13N	05.16E	2003	Ouianbijini
M261	Tajaé	14.13N	05.16E	2003	HKP
M262	Tajaé	14.13N	05.16E	2003	Zongo
PE02779	Tajaé	14.13N	05.16E	1976	Zongo
PE02780	Tajaé	14.13N	05.16E	1976	Guerguéra
M014	Taka	13.46N	00.48E	2003	Gnieye
M015	Taka	13.46N	00.48E	2003	Haïni Kiré
M016	Taka	13.46N	00.48E	2003	Kolala
M017	Taka	13.46N	00.48E	2003	Tchinguel
PE02962	Taka	13.46N	00.48E	1976	Tchinguel
PE02963	Taka	13.46N	00.48E	1976	Foulanya
PE02964	Taka	13.46N	00.48E	1976	Gnieye
PE02965	Taka	13.46N	00.48E	1976	Somno
M159	Tamou	12.45N	02.10E	2003	Meawa Zodi
M160	Tamou	12.45N	02.10E	2003	Gommoni
M161	Tamou	12.45N	02.10E	2003	Bakin Guéro
M162	Tamou	12.45N	02.10E	2003	Zongo
M163	Tamou	12.45N	02.10E	2003	Mardjoua
M164	Tamou	12.45N	02.10E	2003	Ali Cheibou
M165	Tamou	12.45N	02.10E	2003	Sarakoua Sounsou
M166	Tamou	12.45N	02.10E	2003	Darankoba
M167	Tamou	12.45N	02.10E	2003	Jan Guéro
M168	Tamou	12.45N	02.10E	2003	Bossari
PE02983	Tamou	12.45N	02.10E	1976	Somno Koïré
PE02984	Tamou	12.45N	02.10E	1976	Haïni Kiré
M280	Tchadoua	13.33N	07.27E	2003	Zanfaroua
M281	Tchadoua	13.33N	07.27E	2003	Meawa
M282	Tchadoua	13.33N	07.27E	2003	HKP
M283	Tchadoua	13.33N	07.27E	2003	P3 Kollo
M284	Tchadoua	13.33N	07.27E	2003	Zongo
M285	Tchadoua	13.33N	07.27E	2003	Dan Kouroungoussaou
M286	Tchadoua	13.33N	07.27E	2003	CIVT
M287	Tchadoua	13.33N	07.27E	2003	Ouianbijini

M288	Tchadoua	13.33N	07.27E	2003	Ankoutess
PE02718	Tchadoua	13.33N	07.27E	1976	Zongo
M044	Tchi-m-Berkaouan	15.12N	03.32E	2003	Bazaomé
M045	Tchi-m-Berkaouan	15.12N	03.32E	2003	Guerguéra
M046	Tchi-m-Berkaouan	15.12N	03.32E	2003	Matam Hatsi
M047	Tchi-m-Berkaouan	15.12N	03.32E	2003	Eka
M048	Tchi-m-Berkaouan	15.12N	03.32E	2003	Ouitchiabiri
M049	Tchi-m-Berkaouan	15.12N	03.32E	2003	Dan Markoye
M050	Tchi-m-Berkaouan	15.12N	03.32E	2003	Kitsandamo
M051	Tchi-m-Berkaouan	15.12N	03.32E	2003	Bakin Iri
M052	Tchi-m-Berkaouan	15.12N	03.32E	2003	Zongo 1
M053	Tchi-m-Berkaouan	15.12N	03.32E	2003	Dan Ayorou
M054	Tchi-m-Berkaouan	15.12N	03.32E	2003	Zongo 2
PE02915	Tchi-m-Berkaouan	15.12N	03.32E	1976	Zongo
PE02916	Tchi-m-Berkaouan	15.12N	03.32E	1976	Bakin Iri
PE02917	Tchi-m-Berkaouan	15.12N	03.32E	1976	Bazaomé
PE02918	Tchi-m-Berkaouan	15.12N	03.32E	1976	Maewa
M155	Tchoudawa	13.20N	02.34E	2003	Tchoumo
M156	Tchoudawa	13.20N	02.34E	2003	Haïni Kiré Koukou
M157	Tchoudawa	13.20N	02.34E	2003	Somno Darankoba
M158	Tchoudawa	13.20N	02.34E	2003	Somno Bi
PE02985	Tchoudawa	13.20N	02.34E	1976	Haïni Kiré
PE02986	Tchoudawa	13.20N	02.34E	1976	Somno
M089	Tenda	11.59N	03.19E	2003	Haïni Kiré Koukou
M090	Tenda	11.59N	03.19E	2003	Foulan Sory
M091	Tenda	11.59N	03.19E	2003	Somno Bi
M092	Tenda	11.59N	03.19E	2003	Haïni Kiré Gazéra
M093	Tenda	11.59N	03.19E	2003	Fono Laké
PE02825	Tenda	11.59N	03.19E	1976	Haïni Kiré
M416	Tirmini	13.47N	08.48E	2003	Dan Eka
M417	Tirmini	13.47N	08.48E	2003	Ba Angouré
M418	Tirmini	13.47N	08.48E	2003	Dan Tiama
M419	Tirmini	13.47N	08.48E	2003	Tamangagi
PE02609	Tirmini	13.47N	08.48E	1976	Tamangagi
PE02610	Tirmini	13.47N	08.48E	1976	Ba Angouré
M033	Tounfalis	14.19N	03.17E	2003	Dan Ayorou
M034	Tounfalis	14.19N	03.17E	2003	Bazaomé
M035	Tounfalis	14.19N	03.17E	2003	Matam Hatsi
M036	Tounfalis	14.19N	03.17E	2003	Zongo
M060	Tounfalis	14.19N	03.17E	2003	Guerguéra
M061	Tounfalis	14.19N	03.17E	2003	Matam Hatsi

M062	Tounfalis	14.19N	03.17E	2003	Dankoutouboul
M063	Tounfalis	14.19N	03.17E	2003	Bakin Iri
M064	Tounfalis	14.19N	03.17E	2003	Eka
PE02901	Tounfalis	14.19N	03.17E	1976	Dan Ayorou
PE02902	Tounfalis	14.19N	03.17E	1976	Zongo
PE02903	Tounfalis	14.19N	03.17E	1976	Bazaomé
M383	Tsatsoumbroum	12.07N	08.29E	2003	Zongo 1
M384	Tsatsoumbroum	12.07N	08.29E	2003	Zongo 2
M385	Tsatsoumbroum	12.07N	08.29E	2003	Dan Barnou
M386	Tsatsoumbroum	12.07N	08.29E	2003	Maewa
M387	Tsatsoumbroum	12.07N	08.29E	2003	Ankoutess
M388	Tsatsoumbroum	12.07N	08.29E	2003	Matam Hatsi
PE02615	Tsatsoumbroum	12.07N	08.29E	1976	Zongo
PE02616	Tsatsoumbroum	12.07N	08.29E	1976	Dan Barnou
PE02617	Tsatsoumbroum	12.07N	08.29E	1976	Ankoutess
M195	Tsernawa	13.54N	05.21E	2003	Bakin Iri
M196	Tsernawa	13.54N	05.21E	2003	Gassama
M197	Tsernawa	13.54N	05.21E	2003	Zongo Takeï
M198	Tsernawa	13.54N	05.21E	2003	Zongo Ouianbijini
M199	Tsernawa	13.54N	05.21E	2003	Guerguéra Kitsandamo
M200	Tsernawa	13.54N	05.21E	2003	Matam Hatsi
M201	Tsernawa	13.54N	05.21E	2003	HKP
M202	Tsernawa	13.54N	05.21E	2003	Zongo Dabki
PE02776	Tsernawa	13.54N	05.21E	1976	Zongo Outchabiri
PE02777	Tsernawa	13.54N	05.21E	1976	Guerguéra
M001	Wanzerbé	14.45N	00.23E	2003	Bondabia
M002	Wanzerbé	14.45N	00.23E	2003	Tchinguel
M003	Wanzerbé	14.45N	00.23E	2003	Kolala
M004	Wanzerbé	14.45N	00.23E	2003	Somno
M005	Wanzerbé	14.45N	00.23E	2003	Foulanya
M006	Wanzerbé	14.45N	00.23E	2003	Gandassan
PE02970	Wanzerbé	14.45N	00.23E	1976	Foulanya
PE02971	Wanzerbé	14.45N	00.23E	1976	Kolala
PE02972	Wanzerbé	14.45N	00.23E	1976	Haïni Kiré
PE02973	Wanzerbé	14.45N	00.23E	1976	Gnieye
M189	Yaya	13.51N	04.44E	2003	Zongo
M190	Yaya	13.51N	04.44E	2003	Bazaomé
M191	Yaya	13.51N	04.44E	2003	Jan Maewa
M192	Yaya	13.51N	04.44E	2003	Bakan Meawa
M193	Yaya	13.51N	04.44E	2003	Guerguéra
M194	Yaya	13.51N	04.44E	2003	Zongo Kollo

PE02772	Yaya	13.51N	04.44E	1976	Guéro
PE02773	Yaya	13.51N	04.44E	1976	Maewa
