# Supplementary material for: Selection for Earlier Flowering Crop Associated with Climatic Variations in the Sahel
Source: PLoS One. 2011 May 4;6(5):e19563. doi: 10.1371/journal.pone.0019563 (PMC3087796; doi:10.1371/journal.pone.0019563)
Supplement: Table S2 — List of varieties found in the same village in 1976 and 2003. (PDF) [file pone.0019563.s009.pdf]

Table S2

Paired varieties from the 2003 and 1976 samples

Varieties were paired based on shared names and for some varities by direct observation in the field.

Accession 2003	Accession 1976	Variety	Town
M371	PE02599	Badendji	Adoumchi
M332	PE02698	Zongo	Ajékoria
M331	PE02699	Ba Angouré	Ajékoria
M250	PE02803	Guerguéra	Azarori
M270	PE02759	Bakin Iri	Bagaroua
M269	PE02760	Guerguéra	Bagaroua
M272	PE02761	Bazaoné	Bagaroua
M274	PE02758	Zongo	Bagaroua
M028	PE02896	Tchoumo	Baleyara
M031	PE02897	Foulanya	Baleyara
M027	PE02895	Haïni Kiré	Baleyara
M020	PE02958	Kolala	Bangio
M021	PE02955	Gnieye	Bangio
M022	PE02956	Gnieye	Bangio
M065	PE02926	Somno	Banimaté
M068	PE02925	Haïni Kiré	Banimaté
M080	PE02935	Tchoumo	Barakan
M082	PE02937	Somno	Barakan
M077	PE02938	Haïni Kiré	Barakan
M098	PE02827	Zongo	Bengou
M094	PE02826	Ouianbijini	Bengou
M096	PE02828	Maewa	Bengou
M352	PE02584	Boudouma	Bosso
M356	PE02588	Moro	Boudoum
M359	PE02605	Fidéoua	Chéri
M360	PE02604	Moro	Chéri
M351	PE02586	Moro	Chétimari
M051	PE02916	Bakin Iri	Chimbarkaouan
M044	PE02917	Bazaomé	Chimbarkaouan
M052	PE02915	Zongo	Chimbarkaouan
M186	PE02891	Tchoumo	Coquièzé Koara
M183	PE02892	Somno	Coquièzé Koara
M057	PE02921	Bakin Iri	Danbanguiro
M058	PE02923	Zongo	Danbanguiro
M059	PE02924	Bazaoné	Danbanguiro
M238	PE02801	Guerguéra	Déoulé

M265	PE02763	Guéro	Djinguiniss
M266	PE02764	Guerguéra	Djinguiniss
M264	PE02765	Bakin Iri	Djinguiniss
M393	PE02646	Tamangagi	Dogo
M395	PE02647	Ba Angouré	Dogo
M116	PE02861	Guerguéra	Dogo Kiria
M115	PE02862	Bakin Iri	Dogo Kiria
M118	PE02859	Maïwa Baki	Dogo Kiria
M219	PE02754	Zongo	Edir
M218	PE02755	Guerguéra	Edir
M129	PE02885	Somno Gaoré	Falwel
M131	PE02887	Somno Bi	Falwel
M132	PE02886	Haïni Kiré	Falwel
M404	PE02664	Tamangagi	Gangara
M406	PE02663	Ankoutess	Gangara
M407	PE02666	Ba Angouré	Gangara
M290	PE02717	Zongo	Gazaoua
M408	PE02658	Ba Angouré	Guézawa
M409	PE02659	Ankoutess	Guézawa
M373	PE02629	Badendji	Guidiguir
M235	PE02790	Guerguéra	Ibohamane
M236	PE02791	Ejeg	Ibohamane
M312	PE02710	Dan Tiama	Kanan Bakaché
M314	PE02711	Ankoutess	Kanan Bakaché
M315	PE02709	Zongo	Kanan Bakaché
M179	PE02951	Tchoumo	Karma
M180	PE02949	Haïni Kiré	Karma
M358	PE02591	Moro	Kélakam
M111	PE02847	Zongo	Kieché
M110	PE02849	Bakin Iri	Kieché
M112	PE02850	Bazaoné	Kieché
M109	PE02852	Guerguéra	Kieché
M151	PE02888	Haïni Kiré Bounga	Kikoudou Koara
M154	PE02889	Somno	Kikoudou Koara
M369	PE02597	Ba Angouré	Kilakinna
M363	PE02603	Moro	Kojiméri
M012	PE02968	Kolala	Kokoro
M011	PE02966	Tchinguel	Kokoro
M230	PE02786	Gassama	Koloma Baba
M231	PE02785	Guerguéra	Koloma Baba
M169	PE02947	Tchoumo	Koné Béry

M340	PE02697	Zongo	Kornaka
M341	PE02696	Ba Angouré	Kornaka
M247	PE02797	Guerguéra	Korofane
M249	PE02798	Ba Angouré	Korofane
M246	PE02799	Zongo	Korofane
M324	PE02795	Guerguéra	Korohane
M325	PE02796	Ba Angouré	Korohane
M104	PE02836	Guéro	Lido
M101	PE02838	Darankoba	Lido
M100	PE02839	Zongo	Lido
M102	PE02840	Bazaoné	Lido
M275	PE02685	Dan Kazagui	Madarounfa
M277	PE02683	Zongo	Madarounfa
M279	PE02684	Zanfaroua	Madarounfa
M085	PE02816	Haïni Kiré	Margou
M086	PE02817	Somno	Margou
M308	PE02712	Zongo	May Guizawa
M306	PE02713	Ankoutess	May Guizawa
M420	PE02715	Zongo	May Jirgui
M397	PE02628	Gamogi	Mirriah
M208	PE02783	Guerguéra	Mogheur
M144	PE02810	Haïni Kiré	Mokko
M353	PE02582	Boudouma	N'Garoua
M354	PE02583	Guissiri	N'Garoua
M344	PE02722	Zongo	Rwafin Wada
M342	PE02723	Matan Hatchi	Rwafin Wada
M400	PE02660	Ankoutess	Sabon Kafi
M402	PE02661	Ba Angouré	Sabon Kafi
M412S	PE02653	Ba Angouré	Sabongari
M039	PE02908	Zongo	Sanam
M041	PE02909	Bazaoné	Sanam
M037	PE02910	Guerguéra	Sanam
M176	PE02979	Somno	Saoura
M387	PE02617	Ankoutess	Sassoumbouroum
M370	PE02598	Bodendji	Sisya
M122	PE02869	Bakin Iri	Soukoukoutan
M126	PE02870	Guerguéra	Soukoukoutan
M124	PE02868	Zongo	Soukoukoutan
M125	PE02872	Bazaoné	Soukoukoutan
M225	PE02748	Guerguéra	Tabalak
M257	PE02780	Guerguéra	Tajae

M262	PE02779	Zongo	Tajae
M017	PE02962	Tchinguel	Taka
M014	PE02964	Gnieyo	Taka
M089	PE02825	Haïni Kiré	Tanda
M284	PE02718	Zongo	Tchadoua
M156	PE02985	Haïni Kiré	Tchoudawa
M158	PE02986	Somno	Tchoudawa
M417	PE02610	Ba Angouré	Tirmini
M419	PE02609	Tamangagi	Tirmini
M033	PE02901	Dan Ayorou	Tounfalis
M034	PE02903	Bazaoné	Tounfalis
M036	PE02902	Zongo	Tounfalis
M199	PE02777	Guerguéra	Tsernawa
M198	PE02776	Zongo	Tsernawa
M377	PE02625	Gamogi	Ouacha
M003	PE02971	Kolala	Wanzerbé
M005	PE02970	Foulanya	Wanzerbé
M191	PE02773	Maewa	Yaya
