# Supplementary material for: Selection for Earlier Flowering Crop Associated with Climatic Variations in the Sahel
Source: PLoS One. 2011 May 4;6(5):e19563. doi: 10.1371/journal.pone.0019563 (PMC3087796; doi:10.1371/journal.pone.0019563)
Supplement: Table S3 — Main characteristics of different varieties. (DOC) [file pone.0019563.s010.doc]

**Table S3. Main characteristics of different varieties.**

| **Variety name** | **Characteristic** | **Distribution** |
| --- | --- | --- |
| Haini kiré | Average flowering time (FF ~65 days), average spike length (~65 cm), high basal tilling (~10 stems) | South-West Niger |
| Maewa | « Late » flowering variety (FF ~75-80 days), long spike (~75 cm), high basal tilling (~10-11 stems), yellow seed | Southern part of the South-West Niger |
| Zongo | Average flowering time (FF ~65 days), very long spike (~90 cm), average basal tilling ( 8-9 stems) | South-Central Niger |
| Guerguera | Average flowering time (FF ~60-65 days), average spike length (~65 cm), small plant (~180 cm), average basal tilling ( 8-9 stems) | South-Central Niger |
| Ba Angoure | Average flowering time (FF ~60-65 days), short spike (~55 cm), average basal tilling (7-8 stems), small plant (~180-185) | South-Eastern Niger |
| Ankoutess | Average flowering time (FF ~60-65 days), short spike (~40 cm), long spike (~3.5 cm), average basal tilling ( 7-8 stems), small plant (~180-185) | South-Eastern Niger |

FF: flowering time from sowing to female flowering. FF of 60-65 days corresponds to a cycle of around 90-95 days from sowing to maturity. FF of 75-80 days corresponds to 120-130 days to maturity. The table lists the main characteristic of the common varieties
